# Frequency of discrepancies in retracted clinical trial reports versus unretracted reports: blinded case-control study

**DOI:** 10.1136/bmj.h4708

**Published:** 2015-09-20

**Authors:** Graham D Cole, Alexandra N Nowbar, Michael Mielewczik, Matthew J Shun-Shin, Darrel P Francis

**Affiliations:** 1 International Centre for Circulatory Health, National Heart and Lung Institute, Imperial College London W2 1LA, UK

## Abstract

**Objectives** To compare the frequency of discrepancies in retracted reports of clinical trials with those in adjacent unretracted reports in the same journal.

**Design** Blinded case-control study.

**Setting** Journals in PubMed.

**Population **50 manuscripts, classified on PubMed as retracted clinical trials, paired with 50 adjacent unretracted manuscripts from the same journals. Reports were randomly selected from PubMed in December 2012, with no restriction on publication date. Controls were the preceding unretracted clinical trial published in the same journal. All traces of retraction were removed. Three scientists, blinded to the retraction status of individual reports, reviewed all 100 trial reports for discrepancies. Discrepancies were pooled and cross checked before being counted into prespecified categories. Only then was the retraction status unblinded for analysis.

**Main outcome measure** Total number of discrepancies (defined as mathematically or logically contradictory statements) in each clinical trial report.

**Results** Of 479 discrepancies found in the 100 trial reports, 348 were in the 50 retracted reports and 131 in the 50 unretracted reports. On average, individual retracted reports had a greater number of discrepancies than unretracted reports (median 4 (interquartile range 2-8.75) *v* 0 (0-5); P<0.001). Papers with a discrepancy were significantly more likely to be retracted than those without a discrepancy (odds ratio 5.7 (95% confidence interval 2.2 to 14.5); P<0.001). In particular, three types of discrepancy arose significantly more frequently in retracted than unretracted reports: factual discrepancies (P=0.002), arithmetical errors (P=0.01), and missed P values (P=0.02). Results from a retrospective analysis indicated that citations and journal impact factor were unlikely to affect the result.

**Conclusions** Discrepancies in published trial reports should no longer be assumed to be unimportant. Scientists, blinded to retraction status and with no specialist skill in the field, identify significantly more discrepancies in retracted than unretracted reports of clinical trials. Discrepancies could be an early and accessible signal of unreliability in clinical trial reports.

## Introduction

Landmark science cannot always be replicated independently.^1^ ^2^ ^3^ Erroneous research is not uncommon^4^ ^5^ and wastes intellectual and financial resources. More importantly, incorrect results may spawn further clinical research that needlessly draws more patients into trials that would not have been initiated had the original research been reported correctly. In some cases, insecure clinical trials can harm patients when doctors implement their findings in good faith.^6^ ^7^ ^8^

In the specialty of bone marrow stem cell therapy for heart disease, for example, readers are faced with a wide spectrum of conflicting effect sizes that conventional meta-analyses have been unable to explain. In this field, we have recently reported that the number of mathematical or logical discrepancies per trial are the strongest determinant of the effect size reported by the trial.^9^ However, currently, such discrepancies are assumed by some journals to be unimportant and not worth highlighting to readers.^10^ Reaction to the identification of hundreds of discrepancies in only one field varied from interest^11^ to criticism that the entire analysis should be “set aside” and that discrepancies should be routinely accepted as insignificant “flubs”.^12^

Although the number of retractions are increasing,^13^ it remains far lower than the rate of erroneous research,^5^ implying that the literature may be burdened by a substantial proportion of findings that are insecure but unretracted and therefore unrecognised. If discrepancies are more common in retracted studies than unretracted studies, they might represent an accessible signal of concern for readers. We therefore investigated whether discrepancies are more prevalent in retracted than adjacent unretracted reports in the same journals.

## Methods

We undertook a blinded case-control study. We identified discrepancies in randomly selected retracted clinical trial reports, using, in each case, the preceding unretracted trial report in the same journal as the control. We used the same journal because this factor has been identified as a major source of variation in retraction rates.^14^ Annotations of retraction were removed, and the studies were presented in random order to three scientists, who were asked to remain blinded to retraction status.

A PubMed search was conducted in December 2012 for the “retracted publication” publication type and limited to clinical trials, with no restriction on publication date. We used a computer random number generator (Microsoft Excel RAND function) to select members of this set until 50 numbers had been selected. For each trial, a paired control trial was also selected (defined as the unretracted clinical trial) in the same journal, whose PubMed accession sequence was immediately preceding the retracted trial. Watermarks of retraction were removed. The resulting 100 trials were given random sequence numbers between 1 and 100. We decided on a study size of 100 trial reports as a manageable number that could be studied by three scientists, given our previous experience examining reports for discrepancies.^9^ The PDF files of each report were presented to three scientists (GDC, ANN, MM), who were unaware of individual retraction status and asked to refrain from finding this out. Each scientist independently identified factual or mathematical discrepancies without recourse to specialist knowledge. 

Candidate discrepancies proposed by each scientist were pooled and duplicate candidates removed. All three scientists, joined by a fourth senior scientist (DPF), then examined all unique candidate discrepancies and gave an opinion on their individual validity as a discrepancy. At this stage, conferring was allowed. Discrepancies were only accepted as valid if agreed as discrepancies by all four scientists. Table 1 shows categories of discrepancy, along with examples.

**Table 1 tbl1:** Categories of discrepancy

Name	Description	Example	Nature of discrepancy
Possible percentages	A percentage of a group of patients that does not match the subset count displayed but could fit a different subset count	23 of 57 (42%) of patients in intervention group were taking an ACE inhibitor^15^	23/57 is 40%, not 42%; 42% of 57 patients could, however, arise from 24 patients
Impossible percentages	A percentage of a group of patients that cannot exist without fractional patients	31.2% of 200 patients had an infarct in the right coronary artery.^16^	Each patient represents 0.5% of the group. Percentages must therefore be multiples of 0.5%, and 31.2% is not
Factual discrepancies	Two statements that cannot both be true	Abstract: Base excess was 1.04 (SD 0.3) in balanced group at baseline.^17^ Results: Base excess was 1.16 (SD 0.3) in balanced group at baseline^17^	Abstract and results are mutually contradictory
Impossible summary statistics	Summary statistics (mean, median, range, standard deviation) that are not possible, based on the data presented	Median ICU stay in unbalanced group is 13 days. ICU stay ranged from 14 to 444 days^17^	Median must lie within range
Arithmetical errors	Arithmetical errors such as subgroups that do not add up to the total parent group, or differences between before and after measurements that do not match the documented change	Three subgroups of size five, five, and six patients received different doses of treatment. Total number treated, 15 patients^18^	The three subgroups add to 16 patients, but the total is said to be 15
Missed P values	Two groups which are significantly different but are implied to be not different (either explicitly or by omission of a symbol when other comparisons are marked)	Baseline ejection fraction in two groups of 29.4 (SD 12.7; n=191) and 36.1 (SD 13.8; n=200) described as comparable^16^	The published data are sufficient to calculate that the two groups are significantly different (P<0.001)

ACE=angiotensin converting enzyme; ICU=intensive care unit; SD=standard deviation.

### Statistical analysis

#### Descriptive statistics

The study was then unblinded and the reports re-paired. Overall discrepancy counts, and overall counts for the different categories, were compared between the 50 retracted and the 50 unretracted reports by the Wilcoxon signed-rank test. Odds ratios and their confidence intervals were calculated for comparisons between retracted and unretracted studies and the presence or absence of discrepancies.^19^

#### Regression analysis

The number of discrepancies between retracted and unretracted based could be driven by an extreme number of discrepancies in some retracted papers. Taking this factor into consideration in a reanalysis, we quantified the association between retraction status and the number of discrepancies by modelling the number of discrepancies using a zero inflated, negative binomial model. We also used this model to consider the effect of retraction status, year of publication, citations of the trial report, and journal impact factor on discrepancy counts. Regression coefficients were presented as incidence rate ratios for the binomial component and odds ratios for the excess zero component.

#### Sensitivity and specificity analysis

We calculated the sensitivity and specificity of detecting retraction for a range of cut-off thresholds of discrepancy count, and the odds ratio for retraction above these thresholds. Statistical analysis was undertaken by use of the R project for statistical computing^20^ ^21^ ^22^ ^23^ ^24^ ^25^ (code shown in web appendix 1), with figures prepared using ggplot2.^26^

### Patient involvement

No patients were involved in setting the research question or the outcome measures, nor were they involved in the design and implementation of the study. There are no plans to involve patients in dissemination.

## Results

### Trial reports

The search yielded 263 retracted reports of clinical trials published between 1983 and 2012, from which 50 were randomly selected. Twenty three (46%) reports were retracted for misconduct, nine (18%) for errors, seven (14%) for plagiarism and five (10%) for duplication. In six (12%) papers, the reason for retraction was not stated or unclear. Web appendix 2 shows the PubMed identification numbers and number of discrepancies found in each report.

Web appendix 3 lists the trial reports and identified discrepancies. To allow readers to appreciate the findings of our study without necessarily seeing the identities of the trials or authors, each trial report is referenced by a code (R1 to R100). Nevertheless, and only to ensure verifiability, the discrepancies can be viewed in the original reports by entering the PubMed IDs in web appendix 2 at www.pubmed.org.

### Overall discrepancy counts

Of 479 discrepancies found in the 100 trial reports, 348 were in the 50 retracted reports and 131 in the 50 unretracted reports. The overall number of discrepancies was 2.7-fold higher for the 50 retracted reports than for the 50 unretracted reports. Individual report discrepancy counts were higher in retracted (median 4 (interquartile range 2-8.75)) than unretracted reports (median 0 (0-5), P<0.001).

**Figure 1:**
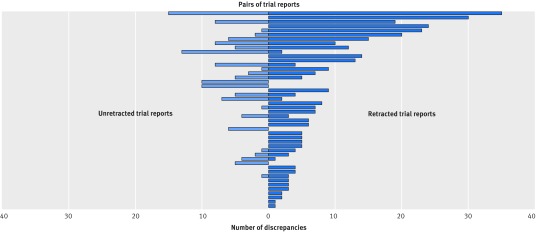
**Fig 1** Discrepancy counts. Paired comparison between retracted reports (right) and unretracted control counterparts (left) for all 100 reports studied. Each bar represents the number of discrepancies in one trial. Pairs are ordered by total number of discrepancies in the pair, with those with the most discrepancies at the bottom

We found discrepancies in 42 (84%) of 50 retracted trials and 24 (48%) of 50 unretracted trials. Of the remaining eight retracted trials with no discrepancies, the reason for retraction was misconduct in four, error in two, and duplication in two. Papers with a discrepancy were significantly more likely to be retracted than those without a discrepancy (odds ratio 5.7 (95% confidence interval 2.2 to 14.5), P<0.001).

#### Regression analysis

We considered the number of discrepancies in trial reports to be a broadly negative binomial distribution but with a certain excess proportion of reports with zero discrepancies. We therefore used a zero inflated, negative binomial regression to investigate the relations between the number of discrepancies and retraction status, year, impact factor of the journal, and number of citations.

Retracted papers were more likely than unretracted papers to have discrepancies, and more of them. In the formal analysis, the number of excess zeros showed a significant relation to retraction status. In this model, retracted reports were less likely than unretracted reports to have excess zero discrepancies (odds ratio 0.14 (95% confidence interval 0.03 to 0.67), P=0.01). No significant association was seen in relation to the year of publication, impact factor, and number of citations (table 2). This same pattern was seen in a univariable analysis (0.11 (0.01 to 0.79), P=0.03).

**Table 2 tbl2:** Zero inflated negative binomial analysis of discrepancies, retraction status, year of publication, journal impact factor, and trial report citations. Only retraction status was significantly associated in both the excess zero components (negatively, in that trial reports are less likely to have zero discrepancies) and binomial components (positively, in that retracted trial reports are more components of the model)

	Incidence rate ratio or odds ratio* (95% CI)	P
**Univariable analysis**		
Binomial component		
Intercept	4.65 (3.03 to 7.15)	<0.001
Retraction	1.62 (0.97 to 2.69)	0.06
Excess zero component		
Intercept	0.78 (0.37 to 1.62)	0.50
Retraction	0.11 (0.01 to 0.79)	0.03
**Multivariable analysis**		
Binomial component		
Intercept	2.45 (0.90 to 6.65)	0.08
Retraction	1.79 (1.07 to 2.99)	0.03
Year	1.05 (0.99 to 1.11)	0.11
Impact factor	0.95 (0.90 to 1.00)	0.07
Citations	1.00 (1.00 to 1.01)	0.24
Excess zero component		
Intercept	4.22 (0.41 to 43.2)	0.23
Retraction	0.14 (0.03 to 0.67)	0.01
Year	0.88 (0.76 to 1.02)	0.08
Impact factor	0.98 (0.83 to 1.16)	0.79
Citations	1.01 (1.00 to 1.01)	0.26

*Incidence rate ratios for binomial components, odds ratios for excess zero components.

Similarly, the number of discrepancies was significantly related to retraction status. Retracted reports had significantly more discrepancies than unretracted reports (incidence rate ratio 1.79 (95% confidence interval 1.07 to 2.99), P=0.03). This same pattern was seen in a univariable analysis (1.62 (0.97 to 2.69), P=0.06). No significant association was seen in relation to the year of publication, impact factor, and number of citations (table 2).

### Types of discrepancy

Some prespecified discrepancy types were significantly more likely to be found in retracted trials than unretracted trials (fig 2). These types were factual discrepancies (median 1 (interquartile range 0-3, range 0-18) *v *median 0 (0-0, 0-11); P=0.002), arithmetical errors (0 (0-0, range 0-2) *v* 0 (0-0, 0-6); P=0.01), and missed P values (0 (0-0, 0-12) *v *0 (0-0, 0-0); P=0.02). For types of discrepancy that did not show a significant difference, the direction of the trend was in each case towards more discrepancies in the retracted trial reports.

**Figure fig2:**
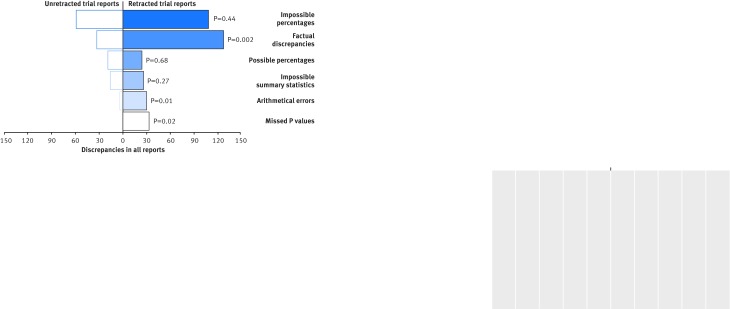
**Fig 2** Total number of discrepancies by type across all 100 trial reports. Each bar represents the total number of one type of discrepancy in 50 trial reports. On the left are those found in unretracted control reports. On the right are those found in retracted reports

### Sensitivity and specificity

We considered whether a particular discrepancy threshold could identify retracted reports by performing a sensitivity-specificity analysis on all 100 trial reports (fig 3). A reader, unaware of retraction status and applying a cut-off point of three or more discrepancies, would have identified retracted papers with 70% sensitivity and 66% specificity (fig 3). The usefulness of this in terms of positive predictive value for identification of problems serious enough to cause retraction will depend on prevalence and will therefore vary. We do not suggest that trial reports be discounted simply based on reaching a threshold number of discrepancies, but rather that the presence of discrepancies might act as a prompt for the authors to provide the community with access to the raw data, in order to secure trust in the result. In our sample, most of the unretracted reports had no discrepancies and 92% had fewer than 10.

**Figure fig3:**
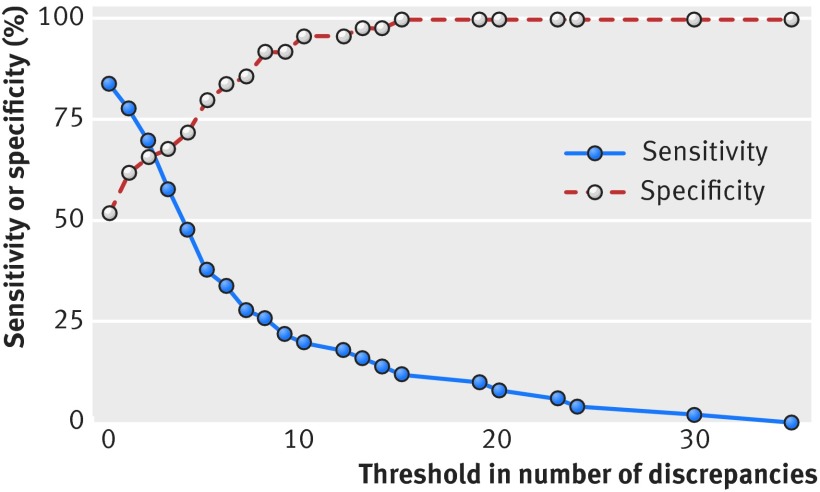
**Fig 3** Sensitivity and specificity of the total number of discrepancies in a trial report exceeding a threshold, and retraction

### Independent identification of discrepancies

Our study design involved three scientists (perhaps simulating reviewers of a manuscript) and one senior scientist (perhaps simulating a final decision maker on publication of a manuscript). For any discrepancy to be considered valid, all four had to agree that there was no viable explanation present in the trial report. Of 479 discrepancies, 299 (62%) were identified by one of the scientists, 78 (16%) were independently identified by two scientists, and 69 (14%) were independently identified by all three scientists. Thirty three (7%) additional discrepancies were noticed by the senior scientist (and subsequently agreed by all others). The time spent by a scientist reading a trial report was available for 269 (90%) of the 300 readings of trial reports (three scientists each reading 100). The median time spent by a scientist on a trial report was 23 minutes (interquartile range 11-38).

### Consideration of potential confounders

We conducted a reanalysis of the following potential confounders that might mediate an association of discrepancies with retraction status:

- Time (because the rate of retraction of literature may have changed over time)
- Citations (because more frequent citation might signify greater scrutiny)
- Journal impact factor (because retraction has been associated with a higher impact factor).

Using a zero inflated negative binomial model, we saw no significant association between any of these potential confounders and the number of discrepancies (table 2).

## Discussion

### Principal findings and implications

This study indicates that the presence of discrepancies in a study report should not be assumed to be meaningless. Discrepancies are significantly more common in retracted rather than unretracted articles.

Peer reviewers and other readers may benefit from this knowledge because it is notoriously difficult for them to evaluate the reliability of a trial’s findings. It is already known^27^ ^28^ ^29^ that the presence of certain features of study design such as blinding, formal enrolment, and automated documentation of results can substantially affect reported effect size. Our study goes beyond this to suggest that identification of discrepancies, even by scientists without particular scientific specialism in the field, might provide an early alarm of unreliability. When doubt exists, it may be practical to repeat some types of scientific study. This is usually not practical for clinical trials, on grounds of time and expense, so there is additional value in readers being able to gauge the reliability of existing reports.

Although the presence of discrepancies seems sensitive to serious problems within trial reports, it is not specific, in that there are many trial reports in good standing with discrepancies. We do not propose that any particular discrepancy threshold should be used as an absolute level for identifying unreliable trial reports. However, it might help to identify trial reports, in which additional documentation from authors might be important to provide reassurance that a study has been reported reliably.

Journals could help in additional ways. Providing a post publication forum for readers to share knowledge of discrepancies is important, because, as our study shows, one reader may spot only a subset of the discrepancies noticed by multiple readers. We believe this finding highlights the difficulty of the task and the likely benefits of crowd sourcing when examining papers after publication. In our study, six retracted trials and seven unretracted trials had a letter to the editor published or a critical editorial raising concerns. Only one of these letters in each group mentioned any discrepancies.

A journal could plan an automatic escalation protocol that would minimise consumption of editorial time. Once it receives a list of discrepancies, it could publish them immediately and request an online supplement of individual patient data from the paper’s authors, if such a supplement was not already provided in the original publication. The journal could publish the time in days and hours from request to receipt. In honestly conducted trials with innocent errors (for example, honest, simple transcription errors), these would be identified as such and quickly corrected. Readers might draw their own conclusions if the dataset is delayed or unavailable.^7^ ^30^

We propose this approach of requesting the raw data for two reasons. Firstly, if the authors were asked to rerun the analyses or present an explanation instead, this could take time to conduct and even more time to achieve agreement between authors. By contrast, the raw data can simply be released by the corresponding author, as there should be no debate. Secondly, such a policy would encourage researchers with nothing to hide to provide the full data as an online supplement in the original publication without waiting for discrepancies to be identified.

Without such protocols and related amendments, journal reviewers and editors must individually find and evaluate the significance of discrepancies. An alternative is that the many eyes of readers could be harnessed in crowd sourced analytical capacity, who would know that their observations contribute to science. Journals could respond at an administrative level without consuming scarce editorial time. Authors would also know that publication would provide genuine scrutiny, not routinely provided or even intended by prepublication peer review.^31^ An alternative mechanism for readers to communicate discrepancies to other readers would be an annotation system such as PubMed Commons or Pubpeer. This circumvents the system of letters to the editor, which is becoming unfit for this purpose because of word count limits and short six week limitation periods in some journals.^10^

Who would do the work of analysing the raw data? Meta-analysts are likely to have time and motivation for this, but so would any reader who wanted to find out the correct answer efficiently. The currently practiced approach, which is to write a letter to the editor, is ineffective. For example, even asking about the registered primary endpoint of a trial,^32^ in which the data seemed to be inexplicably missing from the publication,^33^ may yield an unrevealing reply.^34^ Worse, if the replacement endpoint is different between abstract (and shareholder prospectus^35^) versus individual patient data,^36^ the mathematical impossibility can be stonewalled. Worst of all, statistical experts in the field^37^ can fail to notice this and instead highlight the queries as being “responsibly rebutted.”^38^

### Study limitations

We recognise that even our list of discrepancies (errors) may itself contain errors. Moreover, we have not attempted to establish the mechanism for the discrepancies. We have no way of knowing where each lies on the spectrum from innocent administrative error to intentional fabrication. The strength of our non-judgmental approach is that the presence of discrepancies, rather than any inferred mechanism, is the signal that a trial may be unsound. Author provision of raw data would allow readers to judge the importance of the identified discrepancies and assist appropriate resolution.

We chose our controls to be the preceding clinical trial in the same journal. Our reasoning was that controlling for journal editorial processes, impact factor,^14^ readership, and the journal’s postpublication policy was the priority. Instead of using a fixed protocol to identify the control report, it might have been preferable to use individual judgment to select an unretracted report matched for subject matter. However, we considered that attempting to do this would open the study to bias in such selection. Although our study is not able to confidently state whether the observed pattern is changing over time, each control report was very close in time to its counterpart retracted report.

The overall number of discrepancies (of any type) was significantly different between retracted and unretracted trial reports. For every type of discrepancy, the individual count for that type was numerically higher in retracted than unretracted trial reports; for three types (arithmetical errors, factual discrepancies, and missed P values), this difference between retracted and unretracted reports was statistically significant. This reanalysis that separated the types of discrepancy did not have power to adequately test each type individually. Whether some types of discrepancy are particularly strong markers of trial report unreliability independently therefore remains uncertain. It is also unknown whether some types of discrepancy imply a mechanism (for example, fabrication) and should be considered especially concerning. Alternatively, some readers might be particularly concerned by discrepancies that have immediate therapeutic implications for patients (for example, miscalculations of therapeutic effect size or significance).

In addition, the sample size was constrained by resources because of the time taken to identify, verify, and collate the 479 discrepancies in 100 trial reports, and was not based on a formal power calculation. Future researchers could address other such trial reports or even reassess the trial reports we analysed.

### Misclassified clinical trials—subgroup analysis of pairs of reports meeting a stricter definition of “clinical trial”

During the study, it became apparent that some of the publications identified during the PubMed search would not generally be considered clinical trials of a therapeutic intervention. We performed an additional reanalysis that adopted an aggressive strategy of removing all pairs of clinical trials listed in PubMed where one of the pair was not actually a clinical trial. This reselection process left 68 trial reports in 34 pairs, where both would be generally recognised as clinical trials. Web appendix 4 shows all the figures redrawn for this subgroup. The pattern observed in the 100 trial reports classified as clinical trials in PubMed remained evident in this subgroup of trials with a therapeutic intervention. 

Of 335 discrepancies in the 68 trial reports, 254 were in the 34 retracted reports and 81 were in the 34 unretracted reports. The overall numbers of discrepancies were 3.1-fold higher for the retracted reports than the unretracted reports. Individual discrepancy counts remained higher in retracted reports than unretracted reports (median 5 (interquartile range 3-8.75) *v* 0.5 (0-4.75); P<0.001). 

Thirty two (94%) of 34 retracted reports and 17 (50%) of 34 unretracted reports contained discrepancies. Papers with a discrepancy were significantly more likely to be retracted than those without a discrepancy (odds ratio 16 (95% confidence interval 3.3 to 78), P<0.001). Using zero inflated negative binomial models, results of the univariable and multivariable analyses in this subgroup (web appendix 5) showed a trend similar to the larger dataset, but with wider confidence intervals, owing to a reduced sample size. A threshold of three or more discrepancies showed 76% sensitivity and 68% specificity for identifying retracted reports in this subgroup.

**What is already known on this topic**

Discrepancies (defined as mathematically or logically contradictory statements) can occur in published papers
Whether they matter is disputed, with some experts advising that they be set aside
Some journals will not share discrepancies reported after a fixed time limit or which require more than a certain word limit to communicate

**What this study adds**

Scientists—blinded to retraction status and with no specialist skill the field—identified significantly more discrepancies in retracted than unretracted clinical trial reports
Discrepancies in published clinical trial reports should no longer be assumed to be unimportant and may be an early and accessible signal of unreliability

## Notes

We thank the infrastructural support provided by the National Institute for Health Research Biomedical Research Centre, based at Imperial College Healthcare NHS Trust and Imperial College London.

Ethical approval: Not required.

Data sharing: The complete list of identified discrepancies is shown in web appendix 3. These data are freely available from the corresponding author in editable form on request. We welcome and will make public any corrections or updates from readers. We also provide the raw data in web appendix 6 to permit reanalysis of our data, and the code we used for our analysis in web appendix 1.

The guarantor affirms that this manuscript is an honest, accurate, and transparent account of the study being reported; that no important aspects of the study have been omitted; and that any discrepancies from the study as planned (and, if relevant, registered) have been explained.

## Notes

Cite this as: *BMJ* 2015;351:h4708
