# Supplementary material for: Frequency of discrepancies in retracted clinical trial reports versus unretracted reports: blinded case-control study
Source: BMJ. 2015 Sep 20;351:h4708. doi: 10.1136/bmj.h4708 (PMC4575810; doi:10.1136/bmj.h4708)
Supplement: Supplementary file 1 [file colg020413.ww1_default.pdf]

Web appendix 1

```
library(dplyr)
library(tidyr)
library(ggplot2)
library(pscl)
library(MASS)
library(fmsb)

dprd <- read.csv("./Data/DPR_data.csv")

# Find if both reports classified on pubmed as trials were actually trials (=2 if both, =1 if one, =0 if neither)
dprd <- dprd%>%
    group_by(pair)%>%
    mutate(pair_both_trials=sum(actually_trial))

# Sum total number of discrep for each pair (required if we wanted to plot fig 1 for each type of discrep)
dprd <- dprd%>%
    group_by(pair)%>%
    mutate(pair_discrep=sum(discrep))

# The entire dataset has 100 reports
# On careful examination only 34 pairs (68 in total) were clinical trials of an intervention - these are analysed as a subgroup
dprd_n100 <-dprd
dprd_n68 <- dprd%>%filter(pair_both_trials==2)

# Produce list of discrepancies for basic stats and for sens/spec later on
retracted_discrep <- dprd_n100%>%
  filter(retraction==1)%>%
  dplyr::select(discrep)

unretracted_discrep <- dprd_n100%>%
  filter(retraction==0)%>%
  dplyr::select(discrep)

# Produce list of discrepancies for basic stats and for sens/spec later on for n=68 subgroup
retracted_discrep_n68 <- dprd_n68%>%
  filter(retraction==1)%>%
  dplyr::select(discrep)

unretracted_discrep_n68 <- dprd_n68%>%
  filter(retraction==0)%>%
  dplyr::select(discrep)
```

```
#############
# Basic Stats
#############

# Totals
total_discrep = sum(dprd_n100$discrep)
total_retracted_discrep=sum(retracted_discrep$discrep)
total_unretracted_discrep=sum(unretracted_discrep$discrep)

# Distribution of numbers of discrepancies in reports
table_retracted <- table(retracted_discrep$discrep)
table_unretracted <- table(unretracted_discrep$discrep)

# Non-parametric descriptors

retracted_summary<-summary(retracted_discrep$discrep)
unretracted_summary<-summary(unretracted_discrep$discrep)

# Paired comparison between number of discrepanices in retracted and unretracted
wilcox_data_n100 <- dprd_n100%>%
  dplyr::select(pair, discrep, retraction)%>%
  spread(retraction, discrep)

comparison_retracted_vs_unretracted <- wilcox.test(wilcox_data_n100$"0",
wilcox_data_n100$"1", paired=TRUE)

# Summary stats for different type

summary_types <- dprd_n100%>%
  group_by(retraction)%>%

summarise(median_fd=median(factual_discrepancies),median_ae=median(arithmetic_error
s),median_mpv=median(missed_p_values),min_fd=min(factual_discrepancies),min_ae=min(
arithmetic_errors),min_mpv=min(missed_p_values),max_fd=max(factual_discrepancies),ma
x_ae=max(arithmetic_errors),max_mpv=max(missed_p_values))

#############################
# Basic Stats - n68 subgroup
#############################

# Totals
total_discrep_n68 = sum(dprd_n68$discrep)
total_retracted_discrep_n68=sum(retracted_discrep_n68$discrep)
total_unretracted_discrep_n68=sum(unretracted_discrep_n68$discrep)

# Distribution of numbers of discrepancies in reports
table_retracted_n68 <- table(retracted_discrep_n68$discrep)
```

```
table_unretracted_n68 <- table(unretracted_discrep_n68$discrep)

# Non-parametric descriptors

retracted_summary_n68<-summary(retracted_discrep_n68$discrep)
unretracted_summary_n68<-summary(unretracted_discrep_n68$discrep)

# Paired comparison between number of discrepanices in retracted and unretracted
wilcox_data_n68 <- dprd_n68%>%
  dplyr::select(pair, discrep, retraction)%>%
  spread(retraction, discrep)

comparison_retracted_vs_unretracted_n68 <- wilcox.test(wilcox_data_n68$"0",
wilcox_data_n68$"1", paired=TRUE)

################
###Fig 1 - n=100
################

#get order by total number of discrepancies in pair

discrep_pair_sort_n100 <- dprd_n100%>%
  filter(retraction==1)%>%
  ungroup()%>%
  dplyr::arrange(pair_discrep)

dprd_n100$pair_discrep_sort_code <- factor(dprd_n100$pair,
levels=discrep_pair_sort_n100$pair)

#For the figure we plot the unretracted trials on the left, so we negate the discrepencies
dprd_n100 <- dprd_n100%>%
  mutate(fig_discrep = ifelse(retraction == 0, -discrep, discrep))

p <- ggplot(aes(x=pair_discrep_sort_code, y=fig_discrep, fill=factor(retraction)),
data=dprd_n100)+
  geom_bar(stat="identity", position="identity", width=0.7, color="black", size=0.2)+
  geom_hline(aes(y=0))+
  scale_x_discrete(breaks=NULL)+
  scale_y_continuous(breaks=seq(-40,40,10), limits=c(-40, 40), expand=c(0,0),
labels=abs(seq(-40,40,10)))+
  scale_fill_manual(values=c('white', 'black'), guide=FALSE)+
  coord_flip()+
  labs(y = "Number of Discrepancies")+
  theme(axis.line = element_line(colour = "black"),
      axis.title.x=element_text(vjust=0.4),
      axis.title.y = element_blank(),
      panel.grid.major = element_blank(),
```

```
       panel.grid.minor = element_blank(),
       axis.text.x=element_text(colour="black", size=16),
       axis.title=element_text(size=20),
       panel.background = element_blank())

ggsave("./Figure_1_n100.pdf", plot=p, width=12, height=6)

######################################
###Fig 1 for Online Supplement - n=68
######################################

#get order by total number of discrepancies in pair

discrep_pair_sort_n68 <- dprd_n68%>%
  filter(retraction==1)%>%
  ungroup()%>%
  arrange(pair_discrep)

dprd_n68$pair_discrep_sort_code <- factor(dprd_n68$pair,
levels=discrep_pair_sort_n68$pair)

#For the figure we plot the unretracted trials on the left, so we negate the discrepencies
dprd_n68 <- dprd_n68%>%
 mutate(fig_discrep = ifelse(retraction == 0, -discrep, discrep))

p <- ggplot(aes(x=pair_discrep_sort_code, y=fig_discrep, fill=factor(retraction)),
data=dprd_n68)+
  geom_bar(stat="identity", position="identity", width=0.7, color="black", size=0.2)+
  geom_hline(aes(y=0))+
  scale_x_discrete(breaks=NULL)+
  scale_y_continuous(breaks=seq(-30,30,10), limits=c(-30, 30), expand=c(0,0),
labels=abs(seq(-30,30,10)))+
  scale_fill_manual(values=c('white', 'black'), guide=FALSE)+
  coord_flip()+
  labs(y = "Number of Discrepancies")+
  theme(axis.line = element_line(colour = "black"),
       axis.title.x=element_text(vjust=0.4),
       axis.title.y = element_blank(),
       panel.grid.major = element_blank(),
       panel.grid.minor = element_blank(),
       axis.text.x=element_text(colour="black", size=16),
       axis.title=element_text(size=20),
       panel.background = element_blank())

 ggsave("./Online_Figure_1_n68.pdf", plot=p, width=12, height=6)

###################################
```

```
###Fig 2 - Different Types - n=100
##################################

dprd_types_n100 <- dprd_n100%>%
  group_by(retraction)%>%
  summarise(total_missed_p_values = sum(missed_p_values, na.rm=TRUE),
          total_arithmetic_errors = sum(arithmetic_errors, na.rm=TRUE),
          total_impossible_summary_statistics = sum(impossible_summary_statistics,
na.rm=TRUE),
          total_possible_percentages = sum(possible_percentages, na.rm=TRUE),
          total_factual_discrepancies = sum(factual_discrepancies, na.rm=TRUE),
          total_impossible_percentages = sum(impossible_percentages, na.rm=TRUE))%>%
  gather("type", "discrep", -1)%>%
  mutate(fig_discrep = ifelse(retraction == 0, -discrep, discrep))

p <- ggplot(aes(x=type, y=fig_discrep, alpha=factor(retraction), color=type, fill=type),
data=dprd_types_n100)+
  geom_bar(stat="identity", position="identity")+
  scale_x_discrete(breaks=NULL)+
  scale_y_continuous(breaks=seq(-150,150,30), limits=c(-170, 220), expand=c(0,0),
labels=abs(seq(-150,150,30)))+
  scale_fill_hue(l=45, guide=FALSE)+
  scale_color_hue(l=45, guide=FALSE)+
  geom_hline(aes(y=0))+
  coord_flip()+
  scale_alpha_manual(values=c(0,1), guide=FALSE)+
  labs(x = "Type of Discrepancy", y = "Discrepancies in All Reports")+theme(axis.title.y =
element_text(angle=0))+
  theme(axis.line = element_line(colour = "black"),
        axis.title.x=element_text(vjust=0.4),
        axis.title.y = element_blank(),
        panel.grid.major = element_blank(),
        panel.grid.minor = element_blank(),
        axis.text.x=element_text(colour="black", size=16),
        axis.title=element_text(size=20),
        panel.background = element_blank())

ggsave("./Figure_2_n100.pdf", plot=p, width=12, height=6)

######################################################
###Fig 2 - Different Types - Online Supplement - n=68
######################################################

dprd_types_n68 <- dprd_n68%>%
  group_by(retraction)%>%
  summarise(total_missed_p_values = sum(missed_p_values, na.rm=TRUE),
          total_arithmetic_errors = sum(arithmetic_errors, na.rm=TRUE),
```

```
        total_impossible_summary_statistics = sum(impossible_summary_statistics,
na.rm=TRUE),
        total_possible_percentages = sum(possible_percentages, na.rm=TRUE),
        total_factual_discrepancies = sum(factual_discrepancies, na.rm=TRUE),
        total_impossible_percentages = sum(impossible_percentages, na.rm=TRUE))%>%
  gather("type", "discrep", -1)%>%
  mutate(fig_discrep = ifelse(retraction == 0, -discrep, discrep))

p <- ggplot(aes(x=type, y=fig_discrep, alpha=factor(retraction), color=type, fill=type),
data=dprd_types_n68)+
  geom_bar(stat="identity", position="identity")+
  scale_x_discrete(breaks=NULL)+
  scale_y_continuous(breaks=seq(-120,120,30), limits=c(-140, 190), expand=c(0,0),
labels=abs(seq(-120,120,30)))+
  scale_fill_hue(l=45, guide=FALSE)+
  scale_color_hue(l=45, guide=FALSE)+
  geom_hline(aes(y=0))+
  coord_flip()+
  scale_alpha_manual(values=c(0,1), guide=FALSE)+
  labs(x = "Type of Discrepancy", y = "Discrepancies in All Reports")+theme(axis.title.y =
element_text(angle=0))+
  theme(axis.line = element_line(colour = "black"),
      axis.title.x=element_text(vjust=0.4),
      axis.title.y = element_blank(),
      panel.grid.major = element_blank(),
      panel.grid.minor = element_blank(),
      axis.text.x=element_text(colour="black", size=16),
      axis.title=element_text(size=20),
      panel.background = element_blank())

ggsave("./Online_Figure_2_n68.pdf", plot=p, width=12, height=6)

######################################################
###Plots to visualise data for parametric analysis
######################################################

#Plot of distribution of discrepancies for retracted and unretracted
p_counts <- ggplot(aes(x=discrep), data=dprd)+
  geom_bar()+
  facet_wrap(~retraction)

ggsave("./Xtra_FigX_Counts.pdf", plot=p_counts, width=12, height=6)

#Plot of discrepancies vs year of publication
p_year <- ggplot(aes(x=year, y=discrep),data=dprd_n100)+
  geom_point()+
  facet_wrap(~retraction)
```

```
ggsave("./Xtra_FigX_Year.pdf", plot=p_year, width=12, height=6)

#Plot of discrepancies vs impact factor of journal
p_if <- ggplot(aes(x=if_then, y=discrep), data=dprd_n100)+
  geom_point()+
  facet_wrap(~retraction)

ggsave("./Xtra_FigX_IF.pdf", plot=p_if, width=12, height=6)

#Plot of discrepancies vs citations
p_citations <- ggplot(aes(x=citations, y=discrep), data=dprd_n100)+
  geom_point()+
  facet_wrap(~retraction)

ggsave("./Xtra_FigX_Citations.pdf", plot=p_citations, width=12, height=6)

#Interpretation is that data is zero-inflated,so zero-inflated model should fit better

##################################
### Overall calculation for n=100
##################################

# Model has an exp(), so years (being very large numbers) cause problems.  Hence re-centre
with -1990
dprd_n100$year_base <- dprd_n100$year-1990

# Linear model of discrepancies vs retraction, year, impact factor and citations
dpr_output_n100_glm <- glm(formula = discrep ~ factor(retraction) + year_base + if_then +
citations,
                  family = poisson, data = dprd_n100)

# Zero-inflated Poisson model of discrepancies vs retraction, year, impact factor and
citations
dpr_output_n100_zi <- zeroinfl(discrep ~ factor(retraction) +  year_base + if_then +
citations,
                    data = dprd_n100, dist = "poisson", EM = TRUE)

# Zero-inflated negative binomial model of discrepancies vs retraction, year, impact factor
and citations
dpr_output_n100_zinb <- zeroinfl(discrep ~ factor(retraction) +  year_base + if_then +
citations,
                  data = dprd_n100, dist = "negbin", EM = TRUE)
summary(dpr_output_n100_zinb)
# Decision to use zero-inflated negative binomial model

#Upper, estimate and lower for zero-model
```

```
dprd_upper_zero <-
exp(coef(summary(dpr_output_n100_zinb))$zero[,'Estimate']+1.96*coef(summary(dpr_output_n100_zinb))$zero[,'Std. Error'])
dprd_estimate_zero <- exp(coef(summary(dpr_output_n100_zinb))$zero[,'Estimate'])
dprd_lower_zero <- exp(coef(summary(dpr_output_n100_zinb))$zero[,'Estimate']-
1.96*coef(summary(dpr_output_n100_zinb))$zero[,'Std. Error'])
dprd_zero_summary <-
data.frame(rbind(dprd_lower_zero,dprd_estimate_zero,dprd_upper_zero))

# Interpretation is that retraction is significantly associated with having no discrepancies
# Year, Impact Factor and Citations are not significantly associated
# This is then exported to excel for formatting

#Upper, estimate and lower for count
dprd_upper_count <-
exp(coef(summary(dpr_output_n100_zinb))$count[,'Estimate']+1.96*coef(summary(dpr_output_n100_zinb))$count[,'Std. Error'])
dprd_estimate_count <- exp(coef(summary(dpr_output_n100_zinb))$count[,'Estimate'])
dprd_lower_count <- exp(coef(summary(dpr_output_n100_zinb))$count[,'Estimate']-
1.96*coef(summary(dpr_output_n100_zinb))$count[,'Std. Error'])
dprd_count_summary <-
data.frame(rbind(dprd_lower_count,dprd_estimate_count,dprd_upper_count))

# Interpretation is that, if the report has discrepancies, retraction increases the number of
discrepancies
# Year, Impact Factor and Citations are not significantly associated
# This is then exported to excel for formatting

#Also univariable analysis for retraction vs discrepancies

dpr_output_n100_univariable <- summary(zeroinfl(formula = discrep ~ factor(retraction),
data = dprd_n100, dist = "negbin", EM = TRUE))

###########################################################
###Plots to visualise data for parametric analysis - n=68
###########################################################

# Model has an exp(), so years (being very large numbers) cause problems.  Hence re-centre
with -1990
dprd_n68$year_base <- dprd_n68$year-1990

#Plot of distribution of discrepancies for retracted and unretracted
p_counts_n68 <- ggplot(aes(x=discrep), data=dprd_n68)+
  geom_bar()+
  facet_wrap(~retraction)

ggsave("./Xtra_FigX_Counts_n68.pdf", plot=p_counts_n68, width=12, height=6)
```

```
#Plot of discrepancies vs year of publication
p_year_n68 <- ggplot(aes(x=year, y=discrep),data=dprd_n68)+
  geom_point()+
  facet_wrap(~retraction)

ggsave("./Xtra_FigX_Year_n68.pdf", plot=p_year_n68, width=12, height=6)

#Plot of discrepancies vs impact factor of journal
p_if_n68 <- ggplot(aes(x=if_then, y=discrep), data=dprd_n68)+
  geom_point()+
  facet_wrap(~retraction)

ggsave("./Xtra_FigX_IF_n68.pdf", plot=p_if_n68, width=12, height=6)

#Plot of discrepancies vs citations
p_citations_n68 <- ggplot(aes(x=citations, y=discrep), data=dprd_n68)+
  geom_point()+
  facet_wrap(~retraction)

ggsave("./Xtra_FigX_Citations_n68.pdf", plot=p_citations_n68, width=12, height=6)

###########################################
### Overall calculation for n=68 subgroup
###########################################

# Linear model of discrepancies vs retraction, year, impact factor and citations
dpr_output_n68_glm <- glm(formula = discrep ~ factor(retraction) + year_base + if_then +
citations,
                          family = poisson, data = dprd_n68)

# Zero-inflated Poisson model of discrepancies vs retraction, year, impact factor and
citations
dpr_output_n68_zi <- zeroinfl(discrep ~ factor(retraction) +  year_base + if_then + citations,
                              data = dprd_n68, dist = "poisson", EM = TRUE)

# Zero-inflated negative binomial model of discrepancies vs retraction, year, impact factor
dpr_output_n68_zinb <- zeroinfl(discrep ~ factor(retraction) + year_base,
                               data = dprd_n68, dist = "negbin", EM = TRUE)

# Decision to use zero-inflated negative binomial model

#Upper, estimate and lower for zero-model
dprd_upper_zero_n68 <-
exp(coef(summary(dpr_output_n68_zinb))$zero[,'Estimate']+1.96*coef(summary(dpr_outp
ut_n68_zinb))$zero[,'Std. Error'])
dprd_estimate_zero_n68 <- exp(coef(summary(dpr_output_n68_zinb))$zero[,'Estimate'])
```

```
dprd_lower_zero_n68 <- exp(coef(summary(dpr_output_n68_zinb))$zero[,'Estimate']-
1.96*coef(summary(dpr_output_n68_zinb))$zero[,'Std. Error'])
dprd_zero_summary_n68 <-
data.frame(rbind(dprd_lower_zero_n68,dprd_estimate_zero_n68,dprd_upper_zero_n68))

summary(dpr_output_n68_zinb)
# Interpretation is that very wide confidence intervals mean insufficient data to detect
effect of retraction on excess zeros
# Year is significantly associated with excess zeros on this post-hoc subgroup analysis
# Impact Factor and Citations were removed from analysis (small dataset, risk of overfitting)
# This then exported to excel for formatting

#Upper, estimate and lower for count
dprd_upper_count_n68 <-
exp(coef(summary(dpr_output_n68_zinb))$count[,'Estimate']+1.96*coef(summary(dpr_out
put_n68_zinb))$count[,'Std. Error'])
dprd_estimate_count_n68 <- exp(coef(summary(dpr_output_n68_zinb))$count[,'Estimate'])
dprd_lower_count_n68 <- exp(coef(summary(dpr_output_n68_zinb))$count[,'Estimate']-
1.96*coef(summary(dpr_output_n68_zinb))$count[,'Std. Error'])
dprd_count_summary_n68 <-
data.frame(rbind(dprd_lower_count_n68,dprd_estimate_count_n68,dprd_upper_count_n6
8))
# Interpretation is that, if the report has discrepancies, retraction increases the number of
discrepancies
# Year also significantly associated with discrepancies on this post-hoc subgroup analysis
# Impact Factor and Citations were removed from analysis (small dataset, risk of overfitting)
# This then exported to excel for formatting

dpr_output_n68_univariable <- summary(zeroinfl(formula = discrep ~ factor(retraction),
data = dprd_n68, dist = "negbin", EM = TRUE))

################
# Wilcoxon Below
################

#n100
mww_n100 <- wilcox.test(discrep ~ retraction, data=dprd_n100, paired=TRUE)
mww_fd_n100 <-  wilcox.test(factual_discrepancies ~ retraction, data=dprd_n100,
paired=TRUE)
mww_mpv_n100 <-  wilcox.test(missed_p_values ~ retraction, data=dprd_n100,
paired=TRUE)
mww_iss_n100 <-  wilcox.test(impossible_summary_statistics ~ retraction,
data=dprd_n100, paired=TRUE)
mww_ip_n100 <-  wilcox.test(impossible_percentages ~ retraction, data=dprd_n100,
paired=TRUE)
mww_ip_n100 <-  wilcox.test(impossible_percentages ~ retraction, data=dprd_n100,
paired=TRUE)
```

```
mww_pp_n100 <-  wilcox.test(possible_percentages ~ retraction, data=dprd_n100,
paired=TRUE)
mww_ae_n100 <-  wilcox.test(arithmetic_errors ~ retraction, data=dprd_n100,
paired=TRUE)

# orders change (to match figure order)
n100_types_summary<-
rbind(mww_ip_n100$p.value,mww_fd_n100$p.value,mww_pp_n100$p.value,mww_iss_n1
00$p.value,mww_ae_n100$p.value,mww_mpv_n100$p.value)
#NB this produces summary without names (ie order identifies them relative to Figure 2)

#n68
mww_n68 <- wilcox.test(discrep ~ retraction, data=dprd_n68, paired=TRUE)
mww_ip_n68 <-  wilcox.test(impossible_percentages ~ retraction, data=dprd_n68,
paired=TRUE)
mww_fd_n68 <-  wilcox.test(factual_discrepancies ~ retraction, data=dprd_n68,
paired=TRUE)
mww_pp_n68 <-  wilcox.test(possible_percentages ~ retraction, data=dprd_n68,
paired=TRUE)
mww_iss_n68 <-  wilcox.test(impossible_summary_statistics ~ retraction, data=dprd_n68,
paired=TRUE)
mww_ae_n68 <-  wilcox.test(arithmetic_errors ~ retraction, data=dprd_n68, paired=TRUE)
mww_mpv_n68 <-  wilcox.test(missed_p_values ~ retraction, data=dprd_n68, paired=TRUE)
n68_types_summary<-
rbind(mww_ip_n68$p.value,mww_fd_n68$p.value,mww_pp_n68$p.value,mww_iss_n68$p
.value,mww_ae_n68$p.value,mww_mpv_n68$p.value)

#NB this produces summary without names (ie order identifies them relative to Figure 2)

#########################
#Sens/Spec Graphs - n100
#########################

#Sensitivity = True positives / Total positives (i.e. retracted)
#Specificity = True negatives / total negatives (i.e. not retracted)

threshold <- c(-1,sort(unique(dprd_n100$discrep)))
sens_spec <- data.frame(threshold)

#Define function for point odds ratio estimate

point_OR <- function(true_pos,false_neg,false_pos,true_neg) {
  OR_result_point <- oddsratio(true_pos,false_neg,false_pos,true_neg,0.95)
  OR_result_point <- OR_result_point$estimate
  return(OR_result_point)
}
```

```
#Define function for p-value of odds ratio estimate

p_OR <- function(true_pos,false_neg,false_pos,true_neg) {
  OR_result_p <- oddsratio(true_pos,false_neg,false_pos,true_neg,0.95)
  OR_result_p <- OR_result_p$p.value
  return(OR_result_p)
}

#Define function for lower limit of odds ratio estimate

lower_OR <- function(true_pos,false_neg,false_pos,true_neg) {
  OR_result_lower <- oddsratio(true_pos,false_neg,false_pos,true_neg,0.95)
  OR_result_lower <- OR_result_lower$conf.int[1]
  return(OR_result_lower)
}

#Define function for upper limit of odds ratio estimate

upper_OR <- function(true_pos,false_neg,false_pos,true_neg) {
  OR_result_upper <- oddsratio(true_pos,false_neg,false_pos,true_neg,0.95)
  OR_result_upper <- OR_result_upper$conf.int[2]
  return(OR_result_upper)
}

#Then use these to create new data frame (sens_spec) with odds ratio estimate, upper and
lower values, and p-value, for a number of discrepancies greater than threshold

sens_spec <- sens_spec %>%
  group_by(threshold)%>%
  mutate(true_pos=sum(retracted_discrep$discrep>threshold))%>%
  mutate(true_neg=sum(unretracted_discrep$discrep<=threshold))%>%
  mutate(false_pos=sum(unretracted_discrep$discrep>threshold))%>%
  mutate(false_neg=sum(retracted_discrep$discrep<=threshold))%>%
  mutate(sens=100*true_pos/50)%>%
  mutate(spec=100*true_neg/50)%>%
  # for ROC curve
  mutate(one_minus_spec=100-spec)%>%
  # for odds ratios using functions above
  mutate(OR_result_point=point_OR(true_pos,false_neg,false_pos,true_neg))%>%
  mutate(OR_result_lower=lower_OR(true_pos,false_neg,false_pos,true_neg))%>%
  mutate(OR_result_upper=upper_OR(true_pos,false_neg,false_pos,true_neg))%>%
  mutate(OR_result_p=p_OR(true_pos,false_neg,false_pos,true_neg))

#Graph of sens and spec vs threshold

plt_sens <- ggplot(x=threshold,data=sens_spec)+
  geom_path(aes(x=threshold, y=spec),size=0.75)+
```

```
  geom_point(aes(x=threshold, y=spec),size=2.5)+
  geom_path(aes(x=threshold, y=sens),size=0.75)+
  geom_point(aes(x=threshold, y=sens),size=2.5)+
  scale_x_continuous(lim=c(0,max(sens_spec$threshold)))+
  scale_y_continuous(lim=c(0,100))+
  labs(x = "Threshold")+
  labs(y = "Percentage")+
  theme(axis.line = element_line(colour = "black"),axis.title.y =
element_blank(),panel.grid.major = element_blank(), panel.grid.minor =
element_blank(),axis.text.y=element_text(colour="black", size=16)
,axis.text.x=element_text(colour="black",
size=16),axis.title=element_text(size=20),panel.background = element_blank())+
  theme(axis.title.y = element_text(angle = 0))

ggsave("./Figure_3_Sens_Spec_n100.pdf", plot=plt_sens, width=12, height=6)

#ROC curve

plt.ROC <- ggplot(aes(x=one_minus_spec,y=sens),data=sens_spec)+
  geom_point()+
  geom_path()+
  scale_x_continuous(lim=c(0,100))+
  scale_y_continuous(lim=c(0,100))+
  labs(x = "1-Specificity")+
  labs(y = "Sensitivity")+
  theme_classic()

ggsave("./Xtra_ROC_n100.pdf", plot=plt.ROC, width=6, height=6)

#########################
#Sens/Spec Graphs - n68
#########################

#Sensitivity = True positives / Total positives (i.e. retracted)
#Specificity = True negatives / total negatives (i.e. not retracted)

threshold_n68 <- c(-1,sort(unique(dprd_n68$discrep)))
sens_spec_n68 <- data.frame(threshold_n68)

#Note the funciton is called select(), but as the package MASS also has a select() function it
can
#get masked - so refer to the dplyr select() function as dplyr::select()

retracted_discrep_n68 <- dprd_n68%>%
  filter(retraction==1)%>%
  dplyr::select(discrep)
```

```
unretracted_discrep_n68 <- dprd_n68%>%
  filter(retraction==0)%>%
  dplyr::select(discrep)
```

#Then use these to create new data frame (sens_spec) with odds ratio estimate, upper and lower values, and p-value, for a number of discrepancies greater than threshold

```
sens_spec_n68 <- sens_spec_n68 %>%
  group_by(threshold_n68)%>%
  mutate(true_pos_n68=sum(retracted_discrep_n68$discrep>threshold_n68))%>%
  mutate(true_neg_n68=sum(unretracted_discrep_n68$discrep<=threshold_n68))%>%
  mutate(false_pos_n68=sum(unretracted_discrep_n68$discrep>threshold_n68))%>%
  mutate(false_neg_n68=sum(retracted_discrep_n68$discrep<=threshold_n68))%>%
  mutate(sens_n68=100*true_pos_n68/34)%>%
  mutate(spec_n68=100*true_neg_n68/34)%>%
  # for ROC curve
  mutate(one_minus_spec_n68=100-spec_n68)%>%
  # for simple odds ratio

mutate(OR_result_point_n68=point_OR(true_pos_n68,false_neg_n68,false_pos_n68,true_
neg_n68))%>%

mutate(OR_result_lower_n68=lower_OR(true_pos_n68,false_neg_n68,false_pos_n68,true_
neg_n68))%>%

mutate(OR_result_upper_n68=upper_OR(true_pos_n68,false_neg_n68,false_pos_n68,true
_neg_n68))%>%

mutate(OR_result_p_n68=p_OR(true_pos_n68,false_neg_n68,false_pos_n68,true_neg_n68
))
```

#Graph of sens and spec vs threshold

```
plt_sens_n68 <- ggplot(x=threshold,data=sens_spec_n68)+
  geom_path(aes(x=threshold_n68, y=spec_n68),size=0.75)+
  geom_point(aes(x=threshold_n68, y=spec_n68),size=2.5)+
  geom_path(aes(x=threshold_n68, y=sens_n68),size=0.75)+
  geom_point(aes(x=threshold_n68, y=sens_n68),size=2.5)+
  scale_x_continuous(lim=c(0,30))+
  scale_y_continuous(lim=c(0,100))+
  labs(x = "Threshold")+
  labs(y = "Percentage")+
  theme(axis.line = element_line(colour = "black"),axis.title.y =
element_blank(),panel.grid.major = element_blank(), panel.grid.minor =
element_blank(),axis.text.y=element_text(colour="black", size=16)
,axis.text.x=element_text(colour="black",
size=16),axis.title=element_text(size=20),panel.background = element_blank())+
```

```
  theme(axis.title.y = element_text(angle = 0))

ggsave("./Online_Figure_3__Sens_Spec_n68.pdf", plot=plt_sens_n68, width=12, height=6)

#ROC curve

plt.ROC_n68 <- ggplot(aes(x=one_minus_spec_n68,y=sens_n68),data=sens_spec_n68)+
  geom_point()+
  geom_path()+
  scale_x_continuous(lim=c(0,100))+
  scale_y_continuous(lim=c(0,100))+
  labs(x = "1-Specificity")+
  labs(y = "Sensitivity")+
  theme_classic()

ggsave("./Xtra_ROC_n68.pdf", plot=plt.ROC_n68, width=6, height=6)

##########
###Timings
##########

#Calculate time spent by operators reading trial reports

all_times <- cbind(dprd$op1_mins,dprd$op2_mins, dprd$op3_mins)
all_times <- matrix(all_times, ncol = 1, byrow = TRUE)
all_times_summary <- summary(all_times)
```
