# Supplementary material for: Frequency of discrepancies in retracted clinical trial reports versus unretracted reports: blinded case-control study
Source: BMJ. 2015 Sep 20;351:h4708. doi: 10.1136/bmj.h4708 (PMC4575810; doi:10.1136/bmj.h4708)
Supplement: Supplementary file 2 [file colg020413.ww2_default.pdf]

Web appendix 2

Unretracted Reports			Retracted Reports		
Report Code	PMID	Discrepancies	Report Code	PMID	Discrepancies
R44	18303153	15	R27	18303154	35
R45	22153223	0	R81	22172907	30
R80	20613538	8	R79	20613542	19
R89	10720164	0	R21	10720165	24
R12	18726148	1	R7	22310979	23
R57	19394603	2	R46	19406399	20
R86	18316547	6	R32	18316554	15
R15	12521783	8	R94	12521812	10
R3	15271748	5	R31	15281492	12
R47	20103538	13	R66	20103540	2
R72	19380444	0	R93	19380445	14
R52	20418298	0	R55	20418299	13
R60	19909550	8	R71	20433721	4
R67	18931203	1	R17	18931204	9
R35	16186474	3	R39	16217005	7
R96	11378602	5	R64	11378603	5
R91	8267183	10	R11	8267185	0
R8	10434809	10	R69	10434810	0
R97	21109133	0	R16	21109134	9
R9	16840744	5	R82	16840747	4
R28	16373409	7	R37	16410328	2
R13	12419727	0	R100	12477671	8
R5	12515995	1	R87	14530809	7
R23	17875804	0	R58	17875806	7
R85	19345944	4	R90	19358990	3
R49	17148579	0	R99	17148581	6
R10	15968284	0	R83	15968290	6
R24	18755727	6	R61	18794197	0
R19	10910851	0	R36	10910852	5
R34	18387931	0	R74	18398079	5
R2	7791837	0	R73	7541111	5
R88	12757115	0	R25	12757116	5
R54	19086960	1	R50	19086961	4
R26	17512789	2	R53	17512795	3
R84	16428531	4	R38	16428532	1
R76	17900578	5	R33	17900579	0
R6	10651671	0	R29	10651673	4
R22	10417465	0	R75	10417466	4
R14	17604799	1	R4	17604800	3
R95	18802688	0	R98	18807007	3
R18	18719968	0	R56	18758872	3
R78	15157034	0	R77	15157037	3
R30	7699532	0	R42	7699537	2
R68	17056965	0	R92	17056968	2
R40	18274852	0	R43	18528650	1
R41	9771304	0	R63	9771305	1
R62	10613828	0	R48	10700237	0
R70	10717779	0	R1	10717780	0
R59	9924223	0	R65	9924225	0
R51	15604113	0	R20	15640275	0

Each row consists of a retracted report (right side) and its unretracted counterpart (left side). These pairs of reports are listed in the same order as in Figure 1, namely with the pairs which have the largest number of discrepancies at the top of the list. The publications can be seen, for verification of the discrepancies, by entering the Pubmed Identifier (PMID) at www.pubmed.org.
