# Supplementary material for: Frequency of discrepancies in retracted clinical trial reports versus unretracted reports: blinded case-control study
Source: BMJ. 2015 Sep 20;351:h4708. doi: 10.1136/bmj.h4708 (PMC4575810; doi:10.1136/bmj.h4708)
Supplement: Supplementary file 3 [file colg020413.ww3_default.pdf]

Web appendix 3

Report	Type	Descriptor	Location 1	Detail 1	Location 2	Detail 2
R1		none				
R2		none				
R3	IP	4/106 is not 3.7%	Table 2	4 (3.7%) for diet treatment in the control group. 4/106 is 3.77%. 3/106 is 2.8%. 5/106 is 4.7%.	Abstract	"106 in the control group"
R3	IP	58/106 is not 54.8%	Table 2	58 (54.8%) for insulin treatment in the control group. 58/106 is 54.71%. 57/106 is 53.8%. 59/106 is 55.7%.	Abstract	"106 in the control group"
R3	PP	82/98 is not 87%	Table 2	82 (87%) for general anaesthesia in the study group. 82/98 is 83.7%. 87% is possible but arises from 85/98.	Abstract	"98 in the study [group]"
R3	PP	16/98 is not 13%	Table 2	16 (13%) for regional anaesthesia in the study group. 16/98 is 16.3%. 13% is possible but arises from 13/98.	Abstract	"98 in the study [group]"
R3	PP	58/106 is not 54%	Table 3	58 (54%) for the no insulin, no glucose regimen in the control group. 58/106 is 54.7%. 54% is possible but arises from 57/106.	Abstract	"106 in the control group"
R4	FD	Contradiction over what results are presented in Figure 5 and Figure 8.	Figures 5 and 8	According to the axis description both figures show the incontinence score for the cell group and control group, though with conflicting data.	Figure 8	Figure captions indicate Figure 5 is incontinence score and Figure 8 Quality of life score
R4	FD	Contradiction over number of patients with incontience at follow-up. Reported that only 4 stem cell recipients had incontinence at 1 year of follow-up, but the figure shows 4 with incontinence and 1 with missing value, which indicates that incontenence status unknown.	Results	38/42 patients given autologous cells were continent at one year	Figure 5	1 year follow-up incontinence status missing (unknown) for 40th patient out of 42.
R4	FD	Contradiction over what results are presented in Table 2. Results refer to Table 2 giving outcomes changing more in patients given autologous cells than standard treatment.	Table 2	Comparison is between absolute follow-up values not change		
R5	IP	4% of 43 patients is impossible.	Table 4	Lost to follow up. 2/43 (4%). 1/43 is 2.3%. 2/43 is 4.7%.		
R6		none				
R7	FD	Same symbol used to mean two different quantities without warning.	Results "Changes in All Subjects"	Pre-treatment systolic pressure ± values are SEMs	Results "Divided by Gender"	Female data: corresponding ± values are SDs. No warning for reader that same symbol has different meaning.
R7	FD	Same symbol used to mean two different quantities without warning.	Results "Changes in All Subjects"	Pre-treatment systolic pressure ± values are SEMs	Results "Divided by Gender"	Male data: corresponding ± values are SDs. No warning for reader that same symbol has different meaning.
R7	FD	Same symbol used to mean two different quantities without warning.	Results "Changes in All Subjects"	Pre-treatment diastolic pressure ± values are SEMs	Results "Divided by Gender"	Female data: corresponding ± values are SDs. No warning for reader that same symbol has different meaning.
R7	FD	Same symbol used to mean two different quantities without warning.	Results "Changes in All Subjects"	Pre-treatment diastolic pressure ± values are SEMs	Results "Divided by Gender"	Male data: corresponding ± values are SDs. No warning for reader that same symbol has different meaning.
R7	FD	Same symbol used to mean two different quantities without warning.	Results "Changes in All Subjects"	Post-treatment systolic pressure ± values are SEMs	Results "Divided by Gender"	Female data: corresponding ± values are SDs. No warning for reader that same symbol has different meaning.
R7	FD	Same symbol used to mean two different quantities without warning.	Results "Changes in All Subjects"	Post-treatment systolic pressure ± values are SEMs	Results "Divided by Gender"	Male data: corresponding ± values are SDs. No warning for reader that same symbol has different meaning.
R7	FD	Same symbol used to mean two different quantities without warning.	Results "Changes in All Subjects"	Post-treatment diastolic pressure ± values are SEMs	Results "Divided by Gender"	Female data: corresponding +/- values are SDs. No warning for reader that same symbol has different meaning.
R7	FD	Same symbol used to mean two different quantities without warning.	Results "Changes in All Subjects"	Post-treatment diastolic pressure ± values are SEMs	Results "Divided by Gender"	Male data: corresponding ± values are SDs. No warning for reader that same symbol has different meaning.
R7	FD	Same symbol used to mean two different quantities without warning.	Results "Changes in All Subjects"	Pre-treatment heart rate ± values are SEMs	Results "Divided by Gender"	Female data: corresponding ± values are SDs. No warning for reader that same symbol has different meaning.
R7	FD	Same symbol used to mean two different quantities without warning.	Results "Changes in All Subjects"	Pre-treatment heart rate ± values are SEMs	Results "Divided by Gender"	Male data: corresponding ± values are SDs. No warning for reader that same symbol has different meaning.
R7	FD	Same symbol used to mean two different quantities without warning.	Results "Changes in All Subjects"	Post-treatment heart rate ± values are SEMs	Results "Divided by Gender"	Female data: corresponding ± values are SDs. No warning for reader that same symbol has different meaning.
R7	FD	Same symbol used to mean two different quantities without warning.	Results "Changes in All Subjects"	Post-treatment heart rate ± values are SEMs	Results "Divided by Gender"	Male data: corresponding ± values are SDs. No warning for reader that same symbol has different meaning.
R7	FD	HR_Pre inconsistent with breakdown for males and females	Table 1	Mean 77.904	Table 2	80.97 for 36 females vs 76.110 for 44 males. ((80.97*36)+(76.110*44))/(44+36) = 78.297

R7	ISS	Impossible minimum diastolic BP pre treatment.	Table 1	Minimum 60.770. The fractional part of this cannot be achieved with between 4 and 17 integer measurements.	Methods	Between 4 and 17 treatments, and one measurement per patient, using equipment whose resolution is not less than 1 mmHg, or 1 bpm. Closest achievable is 10/13 which is 0.7692.
R7	ISS	Impossible minimum diastolic BP post treatment.	Table 1	Minimum 61.540. The fractional part of this cannot be achieved with between 4 and 17 integer measurements.	Methods	Closest is 7/13 which is 0.53846...
R7	ISS	Impossible maximum diastolic BP post treatment.	Table 1	Maximum 87.270. The fractional part of this cannot be achieved with between 4 and 17 integer measurements.	Methods	Closest is 3/11 which is 0.2727...
R7	ISS	Impossible minimum heart rate pre treatment	Table 1	Minimum 59.130. The fractional part of this cannot be achieved with between 4 and 17 integer measurements.	Methods	Closest is 2/15 which is 0.1333...
R7	ISS	Impossible maximum heart rate pre treatment	Table 1	Maximum 105.290. The fractional part of this cannot be achieved with between 4 and 17 integer measurements.	Methods	Closest is 5/17 which is 0.2941...
R7	ISS	Impossible minimum heart rate post treatment	Table 1	Minimum 59.710. The fractional part of this cannot be achieved with between 4 and 17 integer measurements.	Methods	Closest is 12/17 which is 0.7059...
R7	ISS	Impossible systolic BP in a male pre treatment	Table 2	Minimum 105.420. The fractional part of this cannot be achieved with between 4 and 17 integer measurements.	Methods	Closest is 5/12 which is 0.4167...
R7	ISS	Impossible diastolic BP in a male post treatment	Table 2	Minimum 65.380. The fractional part of this cannot be achieved with between 4 and 17 integer measurements.	Methods	Closest is 3/8 which is 0.375 exactly
R7	ISS	Impossible heart rate in a female pre treatment	Table 2	Maximum 97.330. The fractional part of this cannot be achieved with between 4 and 17 integer measurements.	Methods	Closest is 2/6 which is 0.3333...
R7	ISS	Impossible heart rate in a female post treatment	Table 2	Minimum 60.360. The fractional part of this cannot be achieved with between 4 and 17 integer measurements.	Methods	Closest is 5/14 which is 0.3571...
R8	ISS	Point estimate is not in middle of confidence interval	Table 3	Onset latency cv for N2O 20% to 40%, interval given as -1.8 to 0.8	Table 3	Point estimate is 0.5
R8	ISS	Point estimate is not in middle of confidence interval	Table 3	Onset latency cv for N2O 20% to 60%, interval given as -1.1 to 2.6	Table 3	Point estimate is -0.8
R8	ISS	Point estimate is outside confidence interval	Table 3	Onset latency cv for N2O 40% to 60%, interval given as -0.2 to 2.4	Table 3	Point estimate is -1.1
R8	ISS	Point estimate is not in middle of confidence interval	Table 3	Amplitude x for N2O 20% to 40%, interval given as-499 to -1	Table 3	Point estimate is -249
R8	ISS	Point estimate is not in middle of confidence interval	Table 3	AUC x for N2O 20% to 40%, interval given as 114 to 2284	Table 3	Point estimate is 1695
R8	ISS	Point estimate is not in middle of confidence interval	Table 3	AUC x for N2O 20% to 60%, interval given as 1278 to 5020	Table 3	Point estimate is 3757
R8	ISS	Point estimate is not in middle of confidence interval	Table 3	AUC x for N2O 40% to 60%, interval given as 960 to 3206	Table 3	Point estimate is 2026
R8	ISS	Point estimate is outside confidence interval	Table 3	Response duration cv for N2O 20% to 60%, interval given as 3.8 to 4.1	Table 3	Point estimate is 0.1
R8	ISS	Point estimate is not in middle of confidence interval	Table 3	Response duration x for N2O 40% to 60%, interval given as -2.3 to 1.7	Table 3	Point estimate is 0.3
R8	ISS	Point estimate is outside confidence interval	Table 3	Onset latency x for N2O 40% vs 60%, latency interval of difference given as 1.3 to 0.8.	Table 3	Point estimate is -0.2
R9	FD	Commonly reported serious adverse events pneumonia in the infliximab subgroups exceeds that in Combined Infliximab (ie all subgroups together)	Table 3	The subgroups include 0 + 12 incidents, while in the combined group only 2 incidences have been listed.		
R9	IP	72.7% of 46 patients is impossible.	Table 1	32(72.7%) of 46 patients receiving Infliximab 3mg/kg had extrapulmonary involvement. 32/46 is 69.6%. 33/46 is 71.7%. 34/46 is 73.9%.		
R9	IP	68.1% of 93 patients is impossible.	Table 1	62(68.1%) of 93 patients receiving Combined Infliximab had extrapulmonary involvement. 62/93 is 66.7%. 63/93 is 67.7%. 64/93 is 68.8%.		
R9	IP	6.7% of 260 patients is impossible.	Table 3	3/260(6.7%) of Infliximab 3mg/kg patients had a reaction through Week 24. 3/260 is 1.2%. 17/260 is 6.5%. 18/260 is 6.9%.		
R9	PP	12/46 patients is not 4.3%.	Table 3	12 (4.3%) of Infliximab 5mg/kg patients had pneumonia. 12/46 patients is 26.1%. 4.3% is possible but arises from 2/46.		
R10		none				
R11		none				
R12	IP	48.2% of 14 patients is impossible.	Table 2	14 patients (1 response, 13 stable) of 29 patients stated to be 48.2%. 13 out of 29 is 44.8%. 14 out of 29 is 48.3%		
R13		none				
R14	PP	2/500 is 0% not 1%.	Table 2	2(1%) of Total Number with Cerebral Palsy have no information available on parental employment.		
R15	FD	Discrepancy in the number of patients in the LEEP group at 12 months visit.	Table V	Total number in LEEP group stated to be 168 patients	Table V	Breakdown of this is: 168 patients with normal outcome, 2 patients with LGSIL and 4 patients with HGSIL. Total is 174 patients.
R15	IP	28.9% of 170 patients is impossible.	Table II	28.9% of cryotherapy group were widowed. 49/170 is 28.8%. 50/170 is 29.4%.		
R15	IP	88.3% of 161 patients is impossible.	Table V	143/161 (88.3%) had normal outcome in cryotherapy group. 142/161 is 88.2%. 143/161 is 88.8%.		
R15	IP	8.0% of 161 patients is impossible.	Table V	12/161 (8.0%) had outcome HGSIL in cryotherapy group. 12/161 is 7.5%. 13/161 is 8.1%.		
R15	ISS	Fisher's exact test calculated incorrectly	Table 4	Values (147, 12; 161, 7) for which Fisher's exact test gives p=0.239	Table 4	States P = 0.379

R15	ISS	Fisher's exact test calculated incorrectly	Table V	Values (143, 18; 168, 6) for which Fisher's exact test gives p=0.0096, Chi2 gives p=0.006 without Yates' correction and 0.011 with Yates correction. Alternate interpretation (143, 18; 162, 6) for which Fisher's exact test gives p=0.0101, Chi2 p=0.0080 without Yates' correction and 0.0147 with Yates' correction	Table V	Stated P = 0.026
R15	PP	126/200 is not 63.5%.	Table I	126/200 (63.5%) had no pain in LEEP group. 126/200 is 63.0%. 63.5% is possible but arises from 127/200.		
R15	PP	168/168 is not 94%.	Table V	168/168 (94%) had normal outcome in LEEP group. 168 out of 168 is 100%. 94% is possible but arises from from158/168.		
R16	FD	Contradiction over upper limit of dural puncture rate	Table 2	Puncture rate ranges from 2.5% (lateral head-down) to 3.7% (sitting)	Discussion	The dural puncture rate was quite high (2.5%-3.4%)
R16	IP	20.1% of 115 patients is impossible	Table 1	20.1% of Sitting position had >1 attempt. 23/115 is 20.0%. 24/115 is 20.9%.		
R16	IP	20.4% of 120 patients is impossible.	Table 1	20.4% of Lateral recumbent head-down group had >1 attempts. 24/120 is 20.0%, 25/120 is 20.8%.		
R16	IP	6.8% of 115 patients is impossible.	Table 2	7/115 (6.8%) of Sitting position had blood in needle. 7/115 is 6.1%, 8/115 is 7.0%.		
R16	IP	3.7% of 115 patients is impossible.	Table 2	4/115 (3.7%) of Sitting position had unintended subarachnoid puncture. 3/115 is 3.5%, 4/115 is 4.3%		
R16	IP	5.35% of 112 patient is impossible.	Table 2	5/112(5.35%) of Lateral recumbent horizontal position had blood in needle. 5/112 is 4.46%. 6/112 is 5.36%.		
R16	IP	2.6% of 112 patients is impossible.	Table 2	3/112(2.6%) of Lateral recumbent horizontal position had unintended subarachnoid puncture. 2/112 is 1.8%. 3/112 is 2.7%.		
R16	IP	4.8% of 120 patients is impossible.	Table 2	5/120(4.8%) of Lateral recumbent head-down position had blood in catheter. 5/120 is 4.2%. 6/120 is 5%.		
R16	PP	14/112 patients is not 11.6%	Table 2	14/112(11.6%) of Lateral recumbent horizontal position had blood in catheter. 14/112 is 12.5%. 11.6% is possible but arises from 13/112.		
R17	FD	Contradiction over whether sCr was higher or lower than normal at baseline.	Results	sCr was slightly higher than normal at baseline in both groups	Figure 2a	Legend says normal sCr<1.3mg/dl. Both groups have mean just below this at baseline.
R17	FD	Text indicates that a Figure shows information (3 month data) but the Figure does not show it.	Results	Three months after discharge from hospital, both, sCr and GFR, almost reached baseline levels (Fig 2).	Figure 2a	Data only provided up to two months.
R17	FD	Contradiction over SD of alpha-GST at baseline	Results	Urine alpha GST had SD of HA 12.7, SD of HES 1.8.	Figure 3	Errors bar size similar for HA and HES groups, instead of being more than 6 fold different in size as would be expected for consistency with stated SDs
R17	FD	>10-fold contradiction over baseline IL-6 values in HA group and unit	Results (page 1500)	HA group baseline IL-6 is 6 pg/dl.	Figure 4a	Mean greater than 10 pg/ml at baseline.
R17	FD	>10-fold contradiction over baseline IL-6 values in HES group and unit	Results (page 1500)	HES group baseline IL-6 is 5 pg/dl.	Figure 4a	Mean greater than 10 pg/ml at baseline.
R17	FD	Contradiction over IL-6 values in HA group after surgery.	Results (page 1500)	HA group after surgery for IL-6 is 180 pg/dl.	Figure 4a	Mean greater being 180 pg/ml = 18000 pg/dl.
R17	FD	Contradiction over IL-6 values in HES group after surgery.	Results (page 1500)	HES group after surgery for IL-6 is 193 pg/dl.	Figure 4a	Mean greater being ca. 193 pg/ml = 19300 pg/dl.
R17	FD	Contradiction over baseline ICAM-1 values in HA group	Results (page 1500)	ICAM-1 plasma levels were slightly increased from normal at baseline	Figure 5	Baseline level in HA group approx 235 (normal range given in legend 200-300ng/ml)
R17	FD	Contradiction over baseline ICAM-1 values in HES group. Text states "slightly increased from normal" but graph shows the mean is slightly below the normal range.	Results (page 1500)	ICAM-1 plasma levels were slightly increased from normal at baseline	Figure 5	Baseline level in HES group approx 195 (normal range given in legend 200-300ng/ml)
R18		none				
R19		none				
R20		none				
R21	AE	Percentages of energy from fat, protein and carbohydrate do not add to 100% for men	Table 1 Legend	Legend states "The values for carbohydrate intake were calculated by subtracting the values for fat and protein intake from those for total energy intake."	Table 1	32.4+14.6+52.4=99.4 (Even allowing for rounding, should be at least 99.9)
R21	AE	Percentages of energy from fat, protein and carbohydrate do not add to 100% for women	Table 1 Legend	Legend states "The values for carbohydrate intake were calculated by subtracting the values for fat and protein intake from those for total energy intake."	Table 1	31.1+13.5+52.5=97.1
R21	AE	Percentages of energy from fat, protein and carbohydrate do not add to 100%	Table 3 Legend	Legend states "The values for carbohydrate intake were calculated by subtracting the values for fat and protein intake from those for total energy intake."	Table 3	24.0%+13.8%+62.4% = 100.2% (Even with rounding, should be no more than 100.1%)
R21	AE	Percentages of energy from fat, protein and carbohydrate do not add to 100%	Table 3 Legend	Legend states "The values for carbohydrate intake were calculated by subtracting the values for fat and protein intake from those for total energy intake."	Table 3	26.0%+13.6%+59.6% = 99.2%
R21	AE	Change in linoleic acid in women incorrectly calculated, even accounting for rounding	Table 5	57.0 to 62.2 is +5.2 not +6.3		
R21	AE	Percentage difference in ApoB for women incorrectly calculated	Table 7	698 to 620 is a percentage change of 78/698 which is 11.1% not 9.0%		
R21	FD	Paired t test described as "parity test"	Table 5			
R21	FD	Discrepancy in the mean for linoleic acid in women on the reference diet	Table 6	Mean given as 23.3	page 173	Mean given as 23.9
R21	FD	Paired t test described as "parity test"	Table 6			
R21	FD	Contradiction over the SE for linoleic acid in women on the reference diet	Table 6	SE given as 1.5	page 173	SE given as 0.7
R21	FD	Contradiction over the mean for linoleic acid in women on the walnut diet	Table 6	Mean given as 29.7	page 173	Mean given as 31.3

R21	FD	Contradiction over the SE for linoleic acid in women on the walnut diet	Table 6	SE given as 0.8	page 173	SE given as 1.4
R21	FD	Contradiction over the mean for linoleic acid in men on the reference diet	Table 6	Mean given as 23.2	page 173	Mean given as 22.5
R21	FD	Contradiction over the SE for linoleic acid in men on the reference diet	Table 6	SE given as 0.7	page 173	SE given as 0.9
R21	FD	Contradiction over the mean for linoleic acid in men on the walnut diet	Table 6	Mean given as 26.2	page 173	Mean given as 30.7
R21	FD	Contradiction over the SE for linoleic acid in men on the walnut diet	Table 6	SE given as 1.4	page 173	SE given as 0.8
R21	FD	Contradiction over the mean for alpha linolenic acid in women on the reference diet	Table 6	Mean given as 0.2	page 173	Mean given as 1.7
R21	FD	Contradiction over the mean for alpha linolenic acid in women on the walnut diet	Table 6	Mean given as 0.3	page 173	Mean given as 3.9
R21	FD	Contradiction over the SE for alpha linolenic acid in women on the reference diet	Table 6	SE given as 0.1	page 173	SE given as 0.2%
R21	FD	Contradiction over the SE for alpha linolenic acid in women on the walnut diet	Table 6	SE given as 0.0	page 173	SE given as 0.4%
R21	FD	Contradiction over the mean for alpha linolenic acid in men on the reference diet	Table 6	Mean given as 0.1	page 173	Mean given as 1.6
R21	FD	Contradiction over the mean for alpha linolenic acid in men on the walnut diet	Table 6	Mean given as 0.2	page 173	Mean given as 3.8
R21	FD	Contradiction over the SE for alpha linolenic acid in men on the reference diet	Table 6	SE given as 0.0	page 173	SE given as 0.1%
R21	FD	Contradiction over the SE for alpha linolenic acid in men on the walnut diet	Table 6	SE given as 0.0	page 173	SE given as 0.4%
R22		none				
R23		none				
R24	FD	Contradiction over % of patients who could remember procedure at 24 hours in midazolam arm	Abstract	59% could recollect. 59% of 22 patients (not possible if 20 patients) is 13 patients, therefore 22-13=9 patients failed to recollect	Results	7/20 (35%) failed to recollect
R24	FD	Contradiction over % of patients who could remember procedure at 24 hours in entonox arm	Abstract	88% could recollect. 88% of 24 patients is 21 patients, therefore 24-21=3 patients failed to recollect.	Results	0% failed to recollect
R24	FD	Contradiction over number of female participants.	Results 1st column	"13 patients were female"	Table 1	12 female
R24	FD	Contradiction over number of patients with previous biopsy.	Results 1st column	53% of patients had previous biopsy (25/46, impossible if n=49)	Table 1	17 patients with previous BMAT
R24	FD	Contradiction over number of patients receiving midazolam, when discussing significant desaturation	Results 2nd column	"4/21 patients (19%) receiving midazolam had significant oxygen desaturation."(Percentage only works for 21)	Table 1	22 patients received midazolam
R24	FD	Contradiction over number of patients receiving midazolam, when discussing flumazenil administration	Results 2nd column	"Flumazenil was administered to 10/21 (48%) patients in the midazolam arm." (Percentage only works for 21 not 22)	Table 1	22 patients received midazolam
R25	MPV	Significant difference between substrate intake in observation period 1 marked as non-significant	Table III	205±13 (n=50) vs 317±60 (n=50) has p=0.0001 but is marked as "NS".		
R25	MPV	Significant difference between substrate intake in observation period 2 marked as non-significant	Table III	208±11 (n=50) vs 315±54 (n=50) has p=0.0001 but is marked as "NS".		
R25	MPV	Significant difference between groups for number of days aspiration in Observation Period 1 marked as non-significant	Table III	0 (n=50) vs 1±1 (n=50) has p=0.0001 but is marked as "NS".		
R25	MPV	Significant difference between groups in number of days pneumonia in Observation Period 1 marked as non-significant	Table III	0 with n=50 vs 2±1 with n=50 has p=0.0001 but is marked as "NS".		
R25	MPV	Significant difference between groups in number of days pneumonia in Observation Period 2 marked as non-significant	Table III	0 with n=50 vs 3±1 with n=50 has p=0.0001 but is marked as "NS".		
R26	FD	Discrepancy in the percentage of Any adverse events related to treatment	Table 2	Any adverse events related to treatment reported as 55.2%	Results (Page 745)	Any adverse events related to treatment reported as 55.7%
R26	IP	55.2% of 309 patients is impossible.	Table 2 any AE, adverse events related to treatment	172/309 (55.2%) had adverse event related to treatment. 170/309 is 55.0%. 171/309 is 55.3%		
R27	FD	Contradiction over number of investigated babies in the table, and the number provided for missing at follow-up or dead	Results page 327	A total of 804 babies of which 72 died and 94 were lost to follow-up: 804-72-94=638	Table 1 and Table 2	Total number of babies undergoing complete examination is given as 639, i.e. 1 more than should be available.
R27	IP	16.8% of 89 patients is impossible.	Results (p327)	16.8% (15/89) with GAs <31 weeks needed treatment. 14/89 is 15.7%. 15/89 is 16.9%.		
R27	IP	10.1% of 186 patients is impossible.	Table 2	17/186 (10.1%) of gestational age 31-32 weeks have mild ROP. 18/186 is 9.7%. 19/186 is 10.2%.		
R27	IP	11.35% of 616 patients is impossible.	Table 4	11.35% of no+mild ROP had maternal oxygen administration. 69/616 is 11.20%. 70/616 is 11.36%.		
R27	IP	12.16% of 616 patients is impossible.	Table 4	12.16% of no+mild ROP had maternal pre-eclampsia. 74/616 is 12.01%. 75/616 is 12.18%.		
R27	IP	7.78% of 616 patients is impossible.	Table 4	7.78% of no+mild ROP had maternal placental abruption. 47/616 is 7.63%. 48/616 is 7.79%.		
R27	IP	10.86% of 616 patients is impossible.	Table 4	10.86% of no+mild ROP had postnatal anaemia. 66/616 is 10.71%. 67/616 is 10.88%.		
R27	IP	4.86% of 616 patients is impossible.	Table 4	4.86% of no+mild ROP had hypoxic ischaemic-encephalopathy. 29/616 is 4.71%. 30/616 is 4.87%.		

R27	IP	15.72% of 616 patients is impossible.	Table 4	15.72% of no+mild ROP had intraventricular haemorrhage. 96/616 is 15.58%. 97/616 is 15.75%.		
R27	IP	24.15% of 616 patients is impossible.	Table 4	24.15% of no+mild ROP had surfactant. 148/616 is 24.03%. 149/616 is 24.19%.		
R27	IP	16.37% of 616 patients is impossible.	Table 4	16.37% of no+mild ROP had respiratory distress syndrome. 100/616 is 16.23%. 101/616 is 16.40%.		
R27	IP	19.45% of 616 patients is impossible.	Table 4	19.45% of no+mild ROP had apnoea >20s. 119/616 is 19.32%. 120/616 is 19.48%.		
R27	IP	4.21% of 616 patients is impossible.	Table 4	4.21% of no+mild ROP had cardiopulmonary resuscitation. 25/616 is 4.06%. 26/616 is 4.22%.		
R27	IP	3.56% of 616 patients is impossible.	Table 4	3.56% of no+mild ROP had blood transfusion. 21/616 is 3.41%. 22/616 is 3.57%.		
R27	IP	34.68% of 616 patients is impossible.	Table 4	34.68% of no+mild ROP had oxygen administration. 213/616 is 34.58%. 214/616 is 34.74%.		
R27	IP	2.43% of 616 patients is impossible.	Table 4	2.43% of no+mild ROP had sepsis. 14/616 is 2.27%. 15/616 is 2.44%.		
R27	IP	7.78% of 616 patients is impossible.	Table 4	7.78% of no+mild ROP had congenital heart disease. 47/616 is 7.63%. 48/616 is 7.79%.		
R27	IP	31.93% of 616 patients is impossible.	Table 4	31.93% of no+mild ROP had hyperbilirubinaemia. 196/616 is 31.82%. 197/616 is 31.98%.		
R27	IP	22.73% of 23 patients is impossible.	Table 4	22.73% of Type 1 ROP had maternal oxygen administration. 5/23 is 21.74%. 6/23 is 26.09%.		
R27	IP	13.64% of 23 patients is impossible.	Table 4	13.64% of Type 1 ROP had maternal pre-eclampsia. 3/23 is 13.04%. 4/23 is 17.39%.		
R27	IP	22.73% of 23 patients is impossible.	Table 4	22.73% of Type 1 ROP had maternal placental abruption. 5/23 is 21.74%. 6/23 is 26.09%.		
R27	IP	45.45% of 23 patients is impossible.	Table 4	45.45% of Type 1 ROP had anaemia. 10/23 is 43.48%. 11/23 is 47.83%.		
R27	IP	22.23% of 23 patients is impossible.	Table 4	22.23% of Type 1 ROP had hypoxic-ischaemic encephalopathy. 5/23 is 21.74%. 6/23 is 26.09%.		
R27	IP	36.36% of 23 patients is impossible.	Table 4	36.36% of Type 1 ROP had intraventricular haemorrhage. 8/23 is 34.78%. 9/23 is 39.13%.		
R27	IP	68.18% of 23 patients is impossible.	Table 4	68.18% of Type 1 ROP had surfactant. 15/23 is 65.22%. 16/23 is 69.57%.		
R27	IP	54.55% of 23 patients is impossible.	Table 4	54.55% of Type 1 ROP had respiratory distress syndrome. 12/23 is 52.17%. 13/23 is 56.52%.		
R27	IP	72.73% of 23 patients is impossible.	Table 4	72.73% of Type 1 ROP had apnoea >20s. 16/23 is 69.57%. 17/23 is 73.91%.		
R27	IP	31.82% of 23 patients is impossible.	Table 4	31.82% of Type 1 ROP had cardiopulmonary resuscitation. 7/23 is 30.43%. 8/23 is 34.78%.		
R27	IP	22.73% of 23 patients is impossible.	Table 4	22.73% of Type 1 ROP had blood transfusion. 5/23 is 21.74%. 6/23 is 26.09%.		
R27	IP	86.36% of 23 patients is impossible.	Table 4	86.36% of Type 1 ROP had oxygen administration. 19/23 is 82.61%. 20/23 is 86.96%.		
R27	IP	4.55% of 23 patients is impossible.	Table 4	4.55% of Type 1 ROP had sepsis. 1/23 is 4.35%. 2/23 is 8.70%.		
R27	IP	9.09% of 23 patients is impossible.	Table 4	9.09% of Type 1 ROP had congenital heart disease. 2/23 is 8.70%. 3/23 is 13.04%.		
R27	IP	50.00% of 23 patients is impossible.	Table 4	50.0% of Type 1 ROP had hyperbilirubinaemia. 11/23 is 47.83%. 12/23 is 52.17%.		
R27	PP	94/804 is not 11.6%	Results (p327)	94 (11.6%) were lost to follow-up. 94/804 is 11.7%. 11.6% is possible but arises from 93/804.		
R27	PP	16/135 is not 11.1%.	Table 1	16/135 (11.1%) of birth weight 1000-1499 have type 1 ROP. 16/135 is 11.9%. 11.1% is possible but arises from 15/135.		
R28	IP	9.5% of 130 patients is impossible.	Table I	9.5% of 130 in HCG group had other indication. 12/130 is 9.2%. 13/130 is 10.0%.		
R28	IP	27.5% of 128 patients is impossible.	Table I	27.5% of 128 in OR group had an unexplained indication for IVF. 35/128 is 27.3%. 36/128 is 28.1%.		
R28	IP	13.5% of 128 patients is impossible.	Table I	13.5% of 128 in OR group had other indication. 17/128 is 13.3%. 18/128 is 14.1%.		
R28	IP	25.4% of 128 patients is impossible.	Table III	25.4% of 128 HCG patients had biochemical outcome. 25.4%. 32/128 is 25.0%. 33/128 is 25.8%.		
R28	IP	23.1% of 128 patients is impossible.	Table III	23.1% of 128 HCG patients had clinical outcome. 29/128 is 22.7%. 30/128 is 23.4%.		
R28	IP	20.8% of 128 patients is impossible.	Table III	20.8% of 128 HCG patients had ongoing outcome. 26/128 is 20.3%. 27/128 is 21.1%.		
R28	IP	20.0% of 128 patients is impossible.	Table III	20.0% of 128 HCG patients had live birth. 25/128 is 19.5%. 26/128 is 20.3%.		
R29	FD	Contradiction over SD of blood loss in hypotension group	Table 1	247 ml	Results	274 ml
R29	FD	Contradiction over mean blood loss in control group	Table 1	1427 ml	Results	1426 ml

R29	ISS	Y-axis does not begin at zero	Figure 2 lower panel			
R29	ISS	Y-axis does not begin at zero	Figure 3 upper panel			
R30		none				
R31	MPV	MPAP significantly different between Transfusion 2 and 10% oxygen groups at 90mins	Table 3	19±4(n=17) vs 25±7(n=17) has p=0.0044		
R31	MPV	SVRI significantly different between Transfusion 1 and Transfusion 2 groups at baseline	Table 3	2116±321(n=16) vs 1774±357(n=17) has p=0.007		
R31	MPV	SVRI significantly different between Transfusion 1 and 100% oxygen groups at baseline	Table 3	2116±321(n=16) vs 1818±331(n=17) has p=0.0134		
R31	MPV	SVRI significantly different between Transfusion 1 and 100% oxygen groups at 30mins	Table 3	1990±347(n=16) vs 1713±259(n=17) has p=0.0138		
R31	MPV	SVRI significantly different between Transfusion 2 and 100% oxygen groups at 30mins	Table 3	2028±425(n=17) vs 1713±259(n=17) has p=0.0137		
R31	MPV	SVRI significantly different between Transfusion 1 and 100% oxygen groups at 60mins	Table 3	2276±331(n=16) vs 1602±347(n=17) has p<0.0001		
R31	MPV	SVRI significantly different between Transfusion 2 and 100% oxygen groups at 60mins	Table 3	2025±365(n=17) vs 1602±347(n=17) has p=0.0015		
R31	MPV	SVRI significantly different between Transfusion 1 and Transfusion 2 groups at 60mins	Table 3	2276±331(n=16) vs 2025±365(n=17) has p=0.0474		
R31	MPV	SVRI significantly different between Transfusion 2 and 100% oxygen groups at 90mins	Table 3	2062±178(n=17) vs 1805±289(n=17) has p=0.0038		
R31	MPV	SVRI significantly different between Transfusion 1 and 100% oxygen groups at 120mins	Table 3	2181±296(n=16) vs 1623±331(n=17) has p<0.0001		
R31	MPV	SVRI significantly different between Transfusion 2 and 100% oxygen groups at 120mins	Table 3	2040±225(n=17) vs 1623±331(n=17) has p=0.0002		
R31	MPV	SVRI significantly different between Transfusion 2 and 100% oxygen groups at 180mins	Table 3	2114±269(n=17) vs 1755±237(n=17) has p=0.0002		
R32	IP	Sensitivity of 40.5% from 46 patients is impossible.	Fig 3	Sensitivity at cut-off A is 40.5%. 18/46 is 39.1%. 19/46 is 41.3%.		
R32	IP	Sensitivity of 64.9% from 46 patients is impossible.	Fig 3	Sensitivity at cut-off B is 64.9%. 29/46 is 63.0%. 30/46 is 65.2%.		
R32	IP	Sensitivity of 73.0 from 46 patients is impossible	Fig 3	Sensitivity at cut-off C is 73.0%. 33/46 is 71.7%. 34/46 is 73.9%.		
R32	IP	Sensitivity of 86.5 from 46 patients is impossible	Fig 3	Sensitivity at cut-off D is 86.5%. 39/46 is 84.8%. 40/46 is 87.0%.		
R32	IP	Sensitivity of 97.3 from 46 patients is impossible	Fig 3	Sensitivity at cut-off E is 97.3%. 44/46 is 95.7%. 45/46 is 97.8%.		
R32	IP	Specificity of 96.0 from 89 patients is impossible	Fig 3	Specificity at cut-off A is 96.0%. 85/89 is 95.6%. 86/89 is 96.6%.		
R32	IP	Specificity of 89.2 from 89 patients is impossible	Fig 3	Specificity at cut-off B is 89.2%. 79/89 is 88.8%. 80/89 is 89.9%.		
R32	IP	Specificity of 85.1 from 89 patients is impossible	Fig 3	Specificity at cut-off C is 85.1%. 75/89 is 84.3%. 76/89 is 85.4%.		
R32	IP	Specificity of 82.4 from 89 patients is impossible	Fig 3	Specificity at cut-off D is 82.4%. 73/89 is 82.0%. 74/89 is 83.1%.		
R32	IP	Specificity of 78.4 from 89 patients is impossible	Fig 3	Specificity at cut-off E is 78.4%. 69/89 is 77.5%. 70/89 is 78.7%.		
R32	IP	Specificity of 48.7 from 89 patients is impossible	Fig 3	Specificity at cut-off F is 48.7%. 43/89 is 48.3%. 44/89 is 49.4%.		
R32	IP	Specificity of 93.6 from 31 patients is impossible.	Fig 4	29/31 is 93.5%. 30/31 is 96.7%.		
R32	IP	19.7% of 31 patients is impossible.	Table 1	3/31(19.7%) have personal cancer history in CRC group. 6/31 is 19.4%. 7/31 is 22.6%.		
R32	IP	95.6% of 23 patients is impossible.	Table 2, colorectal cancer, n (%)	21/23 is 91.3%. 22/23 is 95.7%.		
R32	MPV	Failure to notify readers of p=0.0004 difference in CCSA-2 measurements between two hospitals in the same type of patient.	Table 1	11.98+/-0.55 with n=13 vs 11.08+/-0.46 with n=10 has p=0.0004. Clearly also different in Figure 2.		
R33		none				
R34		none				
R35	AE	Incorrect calculation for sample size.	Methods (Sample Size)	"We estimated that 63 patients were required in each group...". "Assuming a 30% dropout rate we planned to randomize 82 patients to each group." 82 minus 30% is 57.4. If the authors had really started with a		
R35	FD	Contradiction in median change in leg score in BBS<6 group (Baseline Barthel Score<Median) treated with sham acupuncture	Abstract	Subgroup with Baseline Barthel score less than median (median score, 6) in the sham control group had median improvement in leg function 4 points	Figure 2 (2nd from left)	Subgroup with BBS<6 in the sham control group (second column of four) has median 0.
R35	ISS	Median lies outside interquartile range.	Table 1, NIHSS in real acupuncture group	NIHSS median (IQR) = 9 (6-2.3)		
R36	ISS	Imposible SD for raw data presented	Figure 1	Group 1: Range of durations is visibly less than 50 min. Therefore SD must be less than 25*sqrt(25/24) which is 25.52	Text page 380	SD is given as 35 min
R36	ISS	Impossible SD for raw data values presented	Figure 3	Group 1 has a range of tablet counts that has width 4. The SD cannot be larger than (4/2)*sqrt(15/14) which is 2.07	Text page 381	The SD is stated to be 2.3.

R36	ISS	Impossible value for a tablet count in Group 1	Figure 3	Group 1 has 15 members so the median value is that of a single patient, which must be an integer. Figure shows the median to be non-integer.		
R36	ISS	Impossible value for a tablet count in Group 2	Figure 3	Group 2 has 15 members so the median value is that of a single patient, which must be an integer. Figure shows the median to be non-integer.		
R36	ISS	Impossible value for a tablet count in Group 4	Figure 3	Group 4 has 15 members so the median value is that of a single patient, which must be an integer. Figure shows the median to be non-integer.		
R37	MPV	Significant difference between groups for calcium intake	Table 1	523±50 vs 390±65 (both with n=25) has p=0.0001		
R37	MPV	Significant difference between groups for Vitamin D intake	Table 1	315±45 vs 255±75 (both with n=25) has p=0.0012		
R38	AE	Subsets for number of procedures greater than total in group	Table 3	n=19 in Sevoflurane group	Table 3	4 Tonsillectomy + 11 Adenectomy + 5 Tonsillectomy and Adenectomy = 20
R39	MPV	Missed p-value: age in alive significantly lower than CHD group	Table 1	47± (n=6074) vs 51±4 (n=1565) has p<0.0001		
R39	MPV	Missed that age in alive significantly lower than Stroke group.	Table 1	47±5 (n=6074) vs 52±5 (n=708) has p<0.0001		
R39	MPV	Missed that age in alive significantly lower than Other Cardiovascular group.	Table 1	47±5 (n=6074) vs 52±5 (n=172) has p<0.0001		
R39	MPV	Missed that systolic BP in alive significantly lower than Other Cardiovascular group.	Table 1	135±18 (n=6074) vs 140±22 (n=172) has p<0.001		
R39	MPV	Missed that smokers significantly more likely amongst stroke group	Table 1	61% of 708 gives 432/708 smokers in stroke. 56% of 6074 gives 3401/6074 smokers in alive. P value for Fisher's exact test for (432, 276, 3401, 2673) is 0.0116.		
R39	MPV	Missed that inactive significantly more likely amongst CHD group than alive	Table 1	25% of 6074 gives 1519/6074 inactive in alive. 29% of 1565 gives 454/1565 inactive in CHD. P value for Fisher's exact test for (1519, 4555, 454, 1111) is 0.0015		
R39	MPV	Missed that inactive significantly more likely amongst stroke group	Table 1	25% of 6074 gives 1519/6074 inactive in alive. 28% of 708 gives 205/708 inactive in Stroke. P value for Fisher's exact test for (1519, 4555, 205, 503) is 0.0254		
R40		none				
R41		none				
R42	FD	Contradiction over cost per infant of intervention minus startup costs	Results (page 585)	Cost of tracking system minus startup costs was $13.78	Results page 585	Costs include (1) coordinator's salary and fringe benefits $12.77 (2) ongoing supplies $1.11 and (3) startup items $4.17. Total of (1) (2) (ie without startup costs is) is already $13.88.
R42	PP	68/86 is not 78%.	Results (page 585)	Immunization rate at 2 months of age in control group is 78% (68/86). 78% is possible but arises from 67/86.		
R43	FD	Chemical reaction equation breaches conservation of matter.	Methods	Fe + 3/4 O2 + 3/2 H2O -> Fe(OH)3 + heat + steam		There are only enough Fe, O and H on the left to make one Fe(OH)3. There is no H or O left over to make steam.
R44	FD	Contradiction over odds ratio of 750-999g for ROP	Results	Newborns with birth weights of 750-999 grams were 63.82 times more likely to have ROP.	Table 4	Odds ratio 77.255
R44	FD	Contradiction over odds ratio of 1000-1249g for ROP	Results	"Newborns with birth weights of 1000-1249 grams were 77.26 times more likely to have ROP"	Table 4	Odds ratio 74.37
R44	FD	Contradiction over the number of births and its two exclusive and exhaustive subgroups.	Results page 321	A "total of 22 939 000 newborns"	Results page 322	Of these, it is stated that 22 906 005 were without ROP and 28 011 were with ROP. This does not add up to a total of 22 939 000.
R44	FD	Contradiction over whether incidence of haemolytic disease higher in ROP or non-ROP	Results page 322	"All of the comorbidities shown in fig 3 had a significantly higher incidence in ROP newborns compared with non-ROP newborns"	Figure 3 page 322	Haemolytic disease has higher incidence in non-ROP
R44	FD	Contradiction over whether incidence of birth trauma higher in ROP or non-ROP	Results page 322	"All of the comorbidities shown in fig 3 had a significantly higher incidence in ROP newborns compared with non-ROP newborns"	Figure 3 page 322	Birth trauma has higher incidence in non-ROP
R44	FD	Contradiction over whether incidence of intrauterine hypoxia higher in ROP or non-ROP	Results page 322	"All of the comorbidities shown in fig 3 had a significantly higher incidence in ROP newborns compared with non-ROP newborns"	Figure 3 page 322	Intrauterine hypoxia has higher incidence in non-ROP
R44	FD	Contradiction over % of ROP newborns born in teaching hospital	Table 1	77.5% of ROP newborns born in teaching hospital.	Results	78.1% of ROP newborns born in teaching hospital.
R44	FD	Contradiction over LOS for ROP newborns	Table 1	Mean LOS 67.2 days.	Results	Mean LOS 67.7 days.
R44	FD	Contradiction over LOS for non-ROP newborns	Table 1	Mean LOS 2.7 days.	Results	Mean LOS 36.4 days.
R44	FD	Contradiction over hospitalisation charges for ROP (cannot be accounted for by rounding)	Table 1	Mean charges 192664.96	Results	Mean charges 192000
R44	FD	Contradiction over hospitalisation charges for non-ROP	Table 1	Mean charges 3503.32	Results	Mean charges 94096
R44	IP	28.7% of 417 patients is impossible.	Results page 322	Peak frequency in newborns weighing less than 500 grams (119 out of 417 infants or 28.7%). 119/417 is 28.5%. 120/417 is 28.8%.		
R44	IP	11.7% of 747 patients is impossible.	Table 4	Unspecific weight undergoing photocoagulation is 87/747 (11.7%). 87/747 is 11.6%. 88/747 is 11.8%.		
R44	IP	28.7% of 417 patients is impossible.	Table 4	<500g undergoing laser photocoagulation is 119/417 (28.7%). 119/417 is 28.5%. 120/417 is 28.8%.		
R44	ISS	Odds ratio interval for Black babies does not include the odds ratio	Table 4	Odds ratio 0.991 (95 to 1.02)		

R45		none				
R46	AE	Subgroups add up to only 42.7% not 100%.	Table 1	Infertility duration in study group: 15.0%(>=3 years) + 27.7%(<3 years) = 42.7%		
R46	AE	Subgroups add up to more than 100%	Table 1	Myoma location in study group: 59.4% (fundal location) + 41.0% (lower uterine segment) = 100.4%.		
R46	AE	Subgroups add up to only 35.0%.	Table 1	Duration of infertility in control group: 14.0% (>=3 years) + 31.0%(<3 years)=45.0%.		
R46	AE	Subgroups add up to more than 100%.	Table 1	Myoma size in control group: 32.0% (<5mm) + 29.1% (5-10mm) + 22.1% (11-20mm) + 20.4% (>20mm) = 103.6%.		
R46	AE	Subgroups add up to only 99.8%.	Table 1	Myoma type in control group: 58.2% (type 0) + 28.2% (type I) + 13.4% (type II) = 99.8%. Each act of rounding to nearest 0.1% can cause loss of 0.05%. Therefore three values rounded to 0.1% and then totalled could be at most 0.15% below the sum of the original unrounded values which is 100%. Therefore if correctly rounded, the 3 items could never total to less than 99.85%.		
R46	FD	Discrepancy in the number of pregnancies at follow up in the Study group	Results page 726	"64 pregnancies in the study group"	Table 2 and Table 1	Recalculated from the percentages of subgroups with myoma size in table 2 and the percentages of patients in those subgroups given in table 1 there were overall 62 pregnancies in the study group. Using instead myoma count data, the number of pregnancies is 44. Using intead myoma type data, it is 48. Using intead myoma location data, it is 46 or 47.
R46	FD	Contradiction over the number of pregnancies at follow up in the Control group	Results page 726	29 pregnancies occurred in the Control group	Table 2	Recalculated from the percentages of subgroups with myoma size in table II and the percentages of patients in those subgrous given in table 1 there were overall 65 or 66 pregnancies in the control group. Using instead myoma count data, the number of pregnancies is 41. Using instead myoma location data, it is 51.
R46	IP	3.2% of 215 patients is impossible.	Results (Page 726)	7 patients (3.2%) could not undergo in-office hysteroscopic examination. 6/215 is 2.8%. 7/215 is 3.3%.		
R46	IP	15.0% of 101 patients is impossible.	Table 1	15.0% have duration of infertility >=3 years in study group. 15/101 is 14.9%. 16/101 is 15.8%		
R46	IP	41.0% of 101 patients is impossible.	Table 1	41.0% have lower uterine segment location for myoma in study group. 41/101 is 40.6%. 42/101 is 41.6%.		
R46	IP	14.0% of 103 patients is impossible.	Table 1	14.0% have duration of infertility >=3 years in control group. 13/103 is 12.6%. 14/103 is 14.6%.		
R46	IP	31.0% of 103 patients is impossible.	Table 1	31.0% have duration of infertility <3 years in control group. 31/103 is 30.1%. 32/103 is 31.1%.		
R46	IP	22.1% of 103 patients is impossible.	Table 1	22.1% have myoma size 11-20mm in control group. 22/103 is 21.4%. 23/103 is 22.3%.		
R46	IP	58.2% of 103 patients is impossible.	Table 1	58.2% have myoma type 0 in control group. 59/103 is 57.3%. 60/103 is 58.3%.		
R46	IP	13.4% of 103 patients is impossible.	Table 1	13.4% have myoma type II in control group. 13/103 is 12.6%. 14/103 is 13.6%.		
R46	IP	56.2% of 32 patients is impossible.	Table 2	Number of patients with 5-10mm is 32 (31.7% of 101). 18/32 is 56.3%. 17/32 is 53.1%.		
R46	IP	69.6% of 33 patients is impossible.	Table 2	Number of patients with <5mm is 33 (32.0% of 103). 23/33 is 69.7%. 22/33 is 66.7%.		
R46	IP	61.5% of 21 patients is impossible.	Table 2	Number of patients with >20mm is 21 patients (20.4% of 101). 12/21 patients is 57.1%. 13/21 patients is 61.9%.		
R46	IP	36.4% of 10 patients is impossible.	Table 2	Number of patients with >2 myomas is 9.7% of 103=10. 3/10 is 30%. 4/10 is 40%.		
R46	IP	29.0% of 14 patients is impossible.	Table 2	Number of patients with type II myomas is 14 (13.4% of 103). 4/14 is 28.5%. 5/14 is 35.7%.		
R47	FD	Contradictory reasons for exclusion of 3 and 3 patients in group 1 and 2.	Figure 1	6 patients were excluded for protocol violation	Results page 1105	Three patients in each of the groups were excluded. In the parent present and parent absent groups, these included failing to meet inclusion criteria (1 and 1, respectively), missed data collection in the PACU (2 and 1, respectively), and protocol violation (0 and 1, respectively).
R47	IP	3.3% of 149 patients is impossible.	Results: complications (page 1105)	5 patients (3.3%) spontaneously reported feeling faint. 4/147 is 2.7%. 5/147 is 3.4%.		
R47	IP	66.2% of 147 patients is impossible	Table 1	66.2% Use of regional anaesthesia when parent absent. 97/147 is 66.0%. 98/148 is 66.7%.		
R47	IP	2.8% of 147 patients is impossible.	Table 1	2.8% Intravenous maintenance when parent absent. 4/147 is 2.7%. 5/147 is 3.4%.		
R47	IP	63.0% of 147 patients is impossible.	Table 1	63.0% Intraoperative narcotic analgesia when parent absent. 92/147 is 62.6%, 93/147 is 63.3%		
R47	IP	36.9% of 147 patients is impossible.	Table 1	36.9% Previous hospitalization when parent present. 54/147 is 36.7%. 55/147 is 37.4%.		
R47	IP	29.4% of 147 patients is impossible	Table 1	29.4% Previous surgery when parent present. 43/147 is 29.3%. 44/147 is 29.9%.		

R47	IP	23.3% of 147 patients is impossible.	Table 1	23.3% Attendance at preparation program when parent present. 34/147 is 23.1%. 35/147 is 23.8%		
R47	IP	19.1% of 147 patients is impossible.	Table 1	19.1% High socioeconomic class when parent present. 28/147 is 19.0%. 29/147 is 19.7%.		
R47	IP	65.9% of 147 patients is impossible.	Table 1	65.9% Use of regional anaesthesia in parent present group. 96/147 is 65.3%. 97/147 is 66.0%.		
R47	IP	4.3% of 147 patients is impossible.	Table 1	4.3% Intravenous maintenance in parent present group. 6/147 is 4.1%. 7/147 is 4.8%.		
R47	IP	49.3% of 147 patients is impossible.	Table 1	49.3% Intraoperative narcotic analgesia in parent present group. 72/147 is 49.0%. 73/147 is 49.7%.		
R47	IP	45.8% of 134 patients is impossible.	Table 4	45.8% Negative behaviour change at 2wk in parent absent group. 61/134 is 45.5%. 62/134 is 46.2%. Or alternatively 67/147 is 45.6%. 68/147 is 46.3%.	Figure 1	134 in parent absent returned questionnaires
R48		none				
R49		none				
R50	FD	Contradiction over average methylated derivatives in those with myelotoxicity	Results	14500	Table 2	calculated mean 15563 (median 10400)
R50	FD	Contradiction over average 6-TGN in those with myelotoxicity	Results	410	Table 2	calculated mean 451 (median 450)
R50	IP	56.3% of 55 patients is impossible.	Abstract	56.3% did not reach clinical remission. 30/55 is 54.5%. 31/55 is 56.4%.		
R50	IP	Sensitivity of 35% detecting 15 occurrences is impossible.	Results	Cut-off of 11,100 pmol/8 × 10^8 RBC had sensitivity of 35%. 5/15 is 33%. 6/15 is 40%.		
R51		none				
R52		none				
R53	FD	Contradiction over strength of relationship between ADM And AHI	Figure 2a	r=0.428	Table 3	r=0.378
R53	FD	Contradiction over strength of relationship between ADM And ODI	Figure 2b	r=0.658	Table 3	r=0.598
R53	FD	Contradiction over whether ADM is correlated with AHI	Results	Section 3.3 states "Significant correlation is shown between ADM and ODI rather than AHI."	Figure 2a	Figure shows that the ADM-AHI correlation is significant. It states "p<0.01". For r=0.428 and n=48, it is possible to separately calculate the P value to be 0.0024
R54	IP	22.7% of 189 patients is impossible.	Table 2	43/189 (22.7%) had poor global assessment in foam enema group. 42/189 is 22.2%. 43/189 is 22.8%.		
R55	AE	Contradiction over difference in BMI between Groups	Results	The IG had a BMI correspondingly larger by 9.4kg/m^2.	Results	45.88 vs 50.49 kg/m^2. Difference is +4.61 not 9.4
R55	AE	Incorrect calculation of reduction in PEFR in IG	Table 3	Reduction 2.1	Table 3	Preoperative PEFR 6.2, 1 hour postoperative 3.94 = 2.26 (2.205 with most lenient rounding)
R55	AE	Incorrect calculation of reduction in PEFR in SG	Table 3	Reduction 1.6	Table 3	Preoperative PEFR 5.88, 1 day post operative 3.30 = 2.58
R55	FD	Contradiction over significance of difference in FEV1 between SG and ID	Results	1.34L vs 1.84L (p=0.004)	Table 2	p=0.0004
R55	FD	Contradiction over significance of difference in FVC between SG and ID	Results	1.64L vs 2.20L (p=0.0000)	Table 2	p=0.0005
R55	FD	Contradiction over significance of difference in PEFR between SG and ID	Results	3.30L vs 4.70L (p=0.001)	Table 2	p=0.0016 (would round to 0.002)
R55	FD	Contradiction over preoperative FEV1 in IG	Results and Table 3	FEV 2.44	Table 2	FEV 2.45
R55	FD	Contradiction over SG baseline FEV1 standard deviation	Table 2	SD=0.69	Table 3	SD=0.49
R55	FD	Contradiction over SG FVC standard deviation	Table 2	SD=0.82	Table 3	SD=0.54
R55	FD	Contradiction over SG PEFR standard deviation	Table 2	SD=1.68	Table 3	SD=1.10
R55	FD	Contradiction over IG FEV1 standard deviation	Table 2	SD=0.59	Table 3	SD=0.44
R55	FD	Contradiction over IG FVC standard deviation	Table 2	SD=0.69	Table 3	SD=0.48
R55	FD	Contradiction over IG PEFR standard deviation	Table 2	SD=1.18	Table 3	SD=0.95
R56	FD	Contradiction over p-value of port site wound infections comparing two groups	Abstract	p=0.03	Results	p=0.018
R56	FD	Contradiction over number of complications.	Results	Seven complications for an overall incidence of 35%.	Table 2	Total of 6 complications
R56	FD	Contradiction over number of patients with adhesions.	Results	"Out of the early complications, only one was considered to be major for an incidence of 5% (intraabdominal adhesions). One patient with late-presenting (8 months postoperatively) intraabdominal adhesions and consecutive bowel obstruction." i.e. 2 patients with adhesions	Table 2	Only 1 patient with adhesions.
R57	AE	Difference between 19.0 and 34.5 incorrectly calculated	Abstract	19.0mg vs 34.5mg; difference given as -14.0mg.		
R57	IP	14.3% of 30 patients is impossible.	Table 1	9/30 (14.3%) of placebo group had a drain at laparoscopy. 4/30 is 13.3%. 5/30 is 16.7%.		
R58	FD	Contradiction over number of patients in Arm B	Table 1 and Results (page 1807)	98 patients	Table 2	97 patients
R58	FD	Contradiction over number of patients in Arm A	Table 1 and Results (page 1807)	97 patients	Table 2	95 patients

R58	IP	23% of 18 patients is impossible.	Results: Treatment-related toxicity	Adverse events led to discontinuation of thalidomide in 4 patients (4 of 18, 23%). 4/18 is 22%. 5/18 is 28%.		
R58	IP	10% of 95 patients is impossible.	Table 2	9/95 (10%) patients in Arm A have MR 3 months after first transplantation. 9/95 is 9%. 10/95 is 11%.		
R58	PP	9/97 patients is not 10%.	Table 2	9/97 (10%) patients in Arm B have MR 3 months after transplantation. 9/97 is 9%. 10% is possible but arises from 10/97.		
R58	PP	50/95 patients is not 54%.	Table 2	50/95 (54%) patients in Arm A have CR and VGPR after second transplantation. 50/95 is 53%. 54% is possible but arises from 51/95.		
R58	PP	36/95 patients is not 39%.	Table 2	36/95 (39%) in Arm A have PR 6 months after transplantation. 36/95 is 38%. 39% is possible but arises from 37/95.		
R59		none				
R60	IP	40.6% of 30 patients is impossible.	Results (page 5)	40.6% of patients relapsing in first year had mediastinal lymph node metastases. 12/30 is 40%. 13/30 is 43.3%.		
R60	IP	6 patients cannot be 15.9% of any group.	Results (page 5)	6(15.9%) of patients without relapse had well-differentiated tumour. 6/38 is 15.8%. 6/37 is 16.2%.		
R60	IP	24 patients cannot be 63.1% of any group.	Results (page 5)	24(63.1%) without relapse had poorly differentiated tumour. 24/38 is 63.2%. 24/39 is 61.5%.		
R60	IP	14.2% of 14 patients is impossible.	Table 1	2/14(14.2%) of T3 tumours were <2cm from carina. 1/14 is 7.1%. 2/14 is 14.3%.		
R60	IP	2.8% of 70 patients is impossible.	Table 1	2/70(2.8%) of T2 tumours involved visceral pleura. 1/70 is 1.4%. 2/70 is 2.9%.		
R60	IP	21.5% of 14 patients is impossible.	Table 1	3/14(21.5%) of T3 tumours involved parietal pleura. 3/14 is 21.4%. 4/14 is 28.6%.		
R60	IP	14.2% of 14 patients is impossible.	Table 1	2/14(14.2%) of T3 tumours involved mediastinal pleura. 1/14 is 7.1%. 2/14 is 14.3%.		
R60	IP	21.5% of 14 patients is impossible.	Table 1	3/14(21.5%) of T3 tumours involved pericardium. 3/14 is 21.4%. 3/14 is 28.6%.		
R61		none				
R62		none				
R63	AE	Sample size calculation has no relation to the present study.	Text page 765	Paper states "We set alpha=0.05 and beta=0.8, and used a large magnitude of effect (effective size 0.8) to estimate a sufficient sample size. The analysis showed that 30 patients per group would be sufficient." This (on the basis that beta=0.8 actually means power, or 1-beta, is 0.8) would be appropriate for a continuous-variable primary endpoint.	Results	However the primary endpoint, and all other endpoints, are dichotomous. The sample size calculation is meaningless for the present study.
R64	FD	Contradiction over total dose of PTX received per day in each patient	Discussion	"We used1.5mg/kg/hr of PTX, which summed up to approximately 2800 mg of PTX per day in each patient."	Table 1	Average weight 171kg. 1.5mg/kg/h is 36mg/kg for the day. Total average dose 6156mg.
R64	FD	Contradiction over SD of sICAM-1 in control group	Results page 954	sICAM-1 concentrations increased in controls from 50.2±8.2 to 98.2±101.	Figure 1	Error bar approximately 10 wide (similar to treatment arm where sd=9.2)
R64	FD	Contradiction over baseline MEGX results	Results page 954	MEGX testing showed normal values at baseline in both groups	Figure 2 legend	Legend states normal value for MEGX <50. Control arm start at approximately mean=65.
R64	FD	Contradiction over baseline MEGX results	Results page 954	MEGX testing showed normal values at baseline in both groups	Figure 2 legend	Legend states normal value for MEGX<50. Treatment arm starts at approximately mean=65.
R64	ISS	Implausible weight of patients - not presented as bariatric study.	Table 1	Average weight 171kg in pentoxifylline group and 173kg in control group		
R65		none				
R66	ISS	Impossible distribution of Sedation-Agitation Score in celiac group	Table 1	Sedation agitation score 1.8+/-0.3 with n=10. But 10 patients cannot have a sedation-agitation score of 1.8+/-0.3. n=10. If all had score 2, then mean=2 and sd=0. The combination giving smallest sd is 9x2 and 1x1 which gives mean=1.8 and sd=0.42. Any other combinations will have larger sd.	Their reference 24	SAS is integer value from 1 to 7
R66	ISS	Impossible distribution of Sedation-Agitation Score in control group	Table 1	Sedation agitation score 2.0+/-0.2 with n=9. If 9x2, then mean=2 and sd=0. To keep mean=2 need to change to 1x1, 7x2, 1x3 which keeps mean=2 but sd=0.5. Changing more values gives larger sd.	Their reference 24	SAS is integer value from 1 to 7
R67	PP	34/325 is not 11%	Figure 2	34/325 (11%) failed exclusion criteria. 34/325 is 10%. 11%. is possible but arises from between 35 and 37/325.		
R68		none				
R69		none				
R70		none				
R71	AE	Localisation subsets in prep group do not add up to overall group size	Table 1	n=120	Table 1	87 colon + 23 rectum = 110 (not 120)
R71	MPV	Distribution of procedures significantly different between treatment groups	Table 1	Chi2 for (40,50; 9,4; 13,26; 25,15; 33,29) is 10.064 with 4 degrees of freedom, i.e. p=0.039		
R71	MPV	Distribution of stages significantly different between treatment groups	Table 1	Chi2 for (9,25; 52,34; 59,65) is 11.525 with 2 degrees of freedom, i.e. p=0.003		
R71	MPV	Proportion of Manual Anastomosis different between groups	Table 1	70/120 vs 88/124 has p=0.0448 by Fisher Exact test		
R72		none				
R73	FD	Contradiction over median days until platelet count>20,000	Results	Median 12 days	Table 3	Median of individual patient data is 14 days

R73	FD	Contradiction over range of days until platelets >20,000	Results	Range 11-15 days	Table 3	One patient took 17 days
R73	FD	Contradiction over number of patients that received only progenitor cells	Table 1	Six patients	Figure 1 Legend	"The five patients who received only progenitor cells". There were 6. (One died day 14 but had received progenitor cells and would have had monitoring until the date of death)
R73	ISS	Median of all colony-forming cells is incorrect	Table 2	Median of figures is 124,000 not 123,000		
R73	ISS	Median of all progenitor cells is incorrect	Table 2	Median of figure is 87,000 not 88,000		
R74	AE	Sum of parts of RFA group (separated by performance status) exceeds the size of whole group. 8 excess patients.	Table 1	n=100	Table 1	78+15+15=108
R74	FD	Conflicting convention of numerical meaning of "A versus B". In some places it means A using B as a reference, and in others it means B using A as a reference	Results: Tumour response, final sentence	For rate difference, "B" is the reference. "Thirty-three patients (35%) achieved an objective response that was sustained for at least 6 months in the TACE group, 36 (36%) in the RFA group, and 52 (54%) in the TACE-RFA group (rate differences: TACE-RFA vs TACE, 0.19 [95% CI, 0.06-0.33;P=.009] and TACE-RFA vs RFA, 0.18 [95% CI, 0.05-0.32;P=.01])."		For Hazard ratio, "A" is the reference, throughout. Eg. "During follow-up, recurrences were detected in 76 patients (80%) in the TACE group, 81 patients (81%) in the RFA group, and 57 patients (59%) in the TACE-RFA group (TACE-RFA vs TACE, HR, 1.82; 95% CI, 1.28-2.58;P=.001 and TACE-RFA vs RFA, HR, 1.90; 95% CI, 1.35-2.68;P.001). "
R74	FD	More TACE patients had variceal bleeding than gastrointestinal haemorrhage. This contradicts the general principle that bleeding and haemorrhage are synonyms, and varices are part of the gastrointestinal tract, and therefore all patients with variceal bleeding must have gastrointestinal haemorrhage.	Table 2	7 patients had variceal bleeding	Table 5	6 Patients had gastrointestinal haemorrhage
R74	FD	More RFA patients had variceal bleeding than gastrointestinal haemorrhage. This contradicts the general principle that bleeding and haemorrhage are synonyms, and varices are part of the gastrointestinal tract, and therefore all patients with variceal bleeding must have gastrointestinal haemorrhage.	Table 2	7 patients had variceal bleeding	Table 5	3 Patients had gastrointestinal haemorrhage
R74	FD	More TACE-RFA patients had variceal bleeding than gastrointestinal haemorrhage. This contradicts the general principle that bleeding and haemorrhage are synonyms, and varices are part of the gastrointestinal tract, and therefore all patients with variceal bleeding must have gastrointestinal haemorrhage.	Table 2	6 patients had variceal bleeding	Table 5	5 Patients had gastrointestinal haemorrhage
R75	FD	Contradiction over number of patients with grade 2 shivering in placebo group.	Table 2	n=5	Results page 697	n=4
R75	MPV	Statistically significant difference between nefopam and clonidine for time in recovery room	Table 2	9.6±3.1 with n=20 vs 99±25 with n=20 has p=0.0001		
R75	MPV	Statistically significant difference between nefopam and placebo for time in recovery room	Table 2	9.6±3.1 with n=20 vs 94±16 with n=20 has p=0.0001		
R75	MPV	Statistically significant difference between nefopam and placebo for heart rate	Table 3	78.0±1.8 with n=20 vs 87.0±12.9 with n=20 has p=0.0037		
R76	IP	20% of 77 patients is impossible.	Table 2	20% of 77 patients in Amana hospital had action line crossed. 15/70 is 19%. 16/70 is 21%.		
R76	IP	85% of 77 patients is impossible.	Table 2	85% of 77 patients in Amana hospital had APGAR recorded as good. 65/77 is 84%. 66/77 is 86%.		
R76	IP	36% of 93 patients is impossible.	Table 2	36% of 93 patients in Muhimbili hospital had substandard cervical dilatation. 33/93 is 35%. 34/93 is 37%.		
R76	PP	Total percentage for condition of baby after birth recorded as "not good" calculated incorrectly.	Table 2	Stated to be 6%. 8% of 77 is 6 and 10% of 93 is 9. 9+6=15. 15/367=4%		
R76	PP	Total percentage for condition of baby after birth recorded as good calculated incorrectly.	Table 2	Stated to be 43%.	Table 2	85% of 77 is not possible but 65 or 66. 11% of 98 is 11. 9% of 99 is 9. 84% of 93 is 78. 65(or 66)+11+9+78=163(or 164). 163(or 164)/367=44%(or 45%).
R77	IP	39% of 48 patients is impossible.	Table 3	Venlafaxine arm, phantom pain. Number of mastectomies is 23+25=48 (from Table 1). 9/48 is not 39%. 18/48 is 38%. 19/48 is 40%.		
R77	PP	10/47 is not 53%.	Table 3	Phantom pain in control group. 10/47 is not 53%. Number of mastectomies is 19+28=47 (from Table 1).		
R77	FD	Incorrect sample size calculation for a 25% relative risk reduction.	Methods	"A power analysis for PMPS as an outcome, with 80% power to detect a 25% reduction in this outcome and a significance of 0.05 or less, assuming 30% incidence of PMPS, indicated that 36 patients were required in each group."		The actual sample size needed for a 25% relative risk reduction from 30% (i.e. to 22.5%) with 80% power and 5% significance threshold is 450 in each group, i.e. more than 10 times more than were actually recruited.
R78		none				
R79	AE	Incorrect calculation of sufficient sample size.	Methods (page 1038)	"Using type I (a) and type II (b) errors of 0.05 and 0.2, respectively, as well as considering a 50% decrease ... and a 30% standard deviation for the serotonin concentration ... minimum sample of 21 patients per group would be necessary."		Number of patients needed would be 7 per group, using standard formulae for an SD/effect size ratio of 30/50, alpha 0.05 and beta 0.2.
R79	AE	Fluid management in Group A is calculated incorrectly	Table 3	5279-1720 is not 3411.		
R79	AE	Fluid management in Group B is calculated incorrectly.	Table 3	5007-1552 is 3455 not 3592		
R79	FD	Contradiction over mean serotonin levels at T1 in the 80% group	Abstract Results	Serotonin level of 80	Results (Page 1038)	Serotonin level of 82.5

R79	FD	Contradiction over SD of serotonin levels at T1 in the 80% group	Abstract Results	SD of 68.2	Results (Page 1038)	SD of 68.4
R79	FD	Contradiction over SD of serotonin levels at T1 in the 30% group	Abstract Results	SD of 130.5	Results (Page 1038)	SD of 130.4
R79	FD	Contradiction over mean serotonin levels at T2 in the 80% group	Abstract Results	Serotonin level of 78.4	Results (Page 1038)	Serotonin level of 80.8
R79	FD	Contradiction over mean serotonin levels at T3 in the 80% group	Abstract Results	Serotonin level of 76.2	Results (Page 1038)	Serotonin level of 78.2
R79	FD	Contradiction of SD of serotonin levels at T3 in the 80% group	Abstract Results	SD of 49.5	Results (Page 1038)	SD of 50.0
R79	FD	Contradiction between text and figures on exactly how many patients were randomized.	Results	30 patients were randomized in the 80% group and 23 patients in the 30% group.	Figure 1	32 patients were randomized to 80% group. 30 patients were randomized to 30% group. Some dropouts occurred AFTER randomization.
R79	FD	~6-fold contradiction in the serotonin concentration at end of surgery in the 30% group	Results (page 1038)	Serotonin concentration of 147.4 ng per 10^9 platelets, i.e. 0.1474 microgram per 10^9 platelets.	Fig 2	Serotonin MEAN concentration of ca. 0.9 microgram = ca. 900 ng per 10^9 platelets.
R79	FD	~3-fold contradiction in the serotonin concentration at end of surgery in the 80% group	Results (page 1038)	Serotonin concentration of 82.5 ng per 10 ^9 platelets --> equals 0.0825 microgram	Fig 2	Serotonin MEAN concentration of > .25 microgram = ca. 250 ng per 10^9 platelets.
R79	FD	~3-fold contradiction in the serotonin concentration at T2 in the 80% group	Results (page 1038)	Serotonin concentration of 80.8 ng per 10 ^9 platelets --> equals 0.0808 microgram	Fig 2	Serotonin MEAN concentration of > .25 microgram = ca. 250 ng per 10^9 platelets.
R79	FD	~3-fold contradiction in the serotonin concentration at T2 in the 30% group	Results (page 1038)	Serotonin concentration of 139.2 ng per 10 ^9 platelets --> equals 0.1392 microgram	Fig 2	Serotonin MEAN concentration of > .5 microgram = ca. 500 ng per 10^9 platelets.
R79	FD	~3-fold contradiction in the serotonin concentration at T3 in the 80% group	Results (page 1038)	Serotonin concentration of 78.2 ng per 10 ^9 platelets --> equals 0.0782 microgram	Fig 2	Serotonin MEAN concentration of > .25 microgram = ca. 250 ng per 10^9 platelets.
R79	FD	~3-fold contradiction in the serotonin concentration at T3 in the 30% group	Results (page 1038)	Serotonin concentration of 124.1 ng per 10 ^9 platelets --> equals 0.1421 microgram	Fig 2	Serotonin MEAN concentration of > .5 microgram = ca. 500 ng per 10^9 platelets.
R79	FD	Contradiction over whether intraoperative fluid management in Group A is +3592 ml or +3411 ml	Table 1	Given as +3592 ml	Table 3	Given as +3411mls
R79	FD	Contradiction over whether intraoperative fluid management in Group B is +3592 ml or +3411 ml	Table 1	Given as +3411 ml	Table 3	Given as +3592 ml
R79	IP	69% of 23 patients is impossible.	Table 2	69% of 23 patients in group B are non-smokers. 15/23 is 65%. 16/23 is 70%.		
R80	IP	29% of 20 patients is impossible.	Table 1	29% of 20 patients in Group 2 taking beta-blocker. 5/20 is 25%. 6/20 is 30%.		
R80	IP	29% of 20 patients is impossible.	Table 1	29% of 20 patients in Group 2 taking ACE inhibitor. 5/20 is 25%. 6/20 is 30%.		
R80	IP	19% of 20 patients is impossible.	Table 1	19% of 20 patients in Group 2 taking diuretic. 3/20 is 15%. 4/20 is 20%.		
R80	IP	76% of 20 patients is impossible.	Table 1	76% of 20 patients in Group 3 with known cardiac disease. 15/20 is 75%. 16/20 is 80%.		
R80	IP	29% of 20 patients is impossible.	Table 1	29% of 20 patients in Group 3 with diabetes. 5/20 is 25%. 6/20 is 30%.		
R80	IP	19% of 20 patients is impossible.	Table 1	19% of 20 patients in Group 3 taking beta blockers. 3/20 is 15%. 4/20 is 20%.		
R80	IP	33% of 20 patients is impossible.	Table 1	33% of 20 patients in Group 3 taking ACE inhibitors. 6/20 is 30%. 7/20is 35%.		
R80	IP	24% of 20 patients is impossible.	Table 1	24% of 20 patients in Group 3 taking diuretics. 4/20 is 20%. 5/20 is 25%.		
R81	AE	Subgroups of 4%, 61% and 33% add up to 98% rather than 100%.	Table 1	%s of ECOG PS in radiotherapy group (4% status 0, 61% status 1, 33% status 2). Even with rounding of up to 0.5%, maximum rounding is <1.5% for three components so minimum sum of %s is >98.5% (ie can't round to 98%).		
R81	AE	Percentage of patient group who are male (44%) and female (37%) add up to 81% rather than 100%.	Table 1	44% of radiotherapy group are male. 37% of radiotherapy group are female.		
R81	AE	Percentage of patient group who are male (55%) and female (61%) add up to 116% rather than 100%.	Table 1	55% of no radiotherapy group are male. 61% of no radiotherapy group are female.		
R81	AE	Subgroups of 21% and 77% add up to 98% rather than 100%.	Table 1	21% are Stage I and 77% are Stage II in no radiotherapy group.		
R81	AE	Subgroups of 8%, 63% and 26% add up to 97% rather than 100%.	Table 1	%s of ECOG PS in no radiotherapy group (8% status 0, 63% status 1, 26% status 2).		
R81	FD	Censure marks on overall survival curve inconsistent with follow-up	Results	Follow-up ranged from 59 to 82 months.	Figure 2	Censure marks occur in overall free survival diagram before 59 months
R81	FD	Duration of survival curves inconsistent with follow-up	Results	Follow-up ranged from 59 to 82 months	Figure 1	Progression-free survival curve extends to ten years
R81	FD	Duration of survival curves inconsistent with follow-up	Results	Follow-up ranged from 59 to 82 months	Figure 2	Overall survival curve extends to ten years
R81	FD	Censure marks on progression-free survival curve inconsistent with follow-up	Results	Follow-up ranged from 59 to 82 months	Figure 1	Censure marks occur in progression-free survival curve before 59 months

R81	FD	Survival in RT arm at 10 years is higher than mathematically possible for that number of deaths	Results	18 of 63 patients died	Abstract	10 year survival is 72%. Enrolment began June 2002, and closed July 2009, so no patient had >7yr 1 mo FU at July 2009 closure. Thus there is no 10-year actuarial survival data. Thus the survival is not actuarial but KM. KM survival is always lower than the 1-deaths/starting number (due to loss to follow-up). This means it 10-year KM survival is <=1-18/63 = 71.4%, i.e. CANNOT be 72%
R81	FD	Survival in control arm at 10 years is lower than mathematically possible for that number of deaths	Results	40 of 61 patients died	Abstract	10 year survival is 31%. (see above) This means it 10-year KM survival is <=1-40/61 = 34.4%, i.e. CANNOT be 31%
R81	FD	Progression free survival in the RT arm at 10 years is higher than mathematically possible for that number of patients	Results	18 patients progressed	Results	10 year progression free survival is 72%. (see above) This means it 10-year KM survival is <=1-18/63 = 71.4%, i.e. CANNOT be 72%
R81	IP	28% of 63 patients is impossible	Results	Overall relapse/progression was observed in 18 patients (28%) in the RT arm. 18/63 is 29%. 17/63 is 27%.		
R81	IP	74% of 63 patients is impossible.	Table 1	46/63 (74%) of radiotherapy patients are Stage II. 46/63 is 73%. 47/63 is 75%.		
R81	IP	80% of 63 patients is impossible.	Table 1	51/63 (80%) of radiotherapy patients are low-intermediate international project index. 51/63 is 81%. 50/63 is 79%.		
R81	IP	93% of 63 patients is impossible.	Table 1	59/63 (93%) of radiotherapy patients had bulky disease. 58/63 is 92%. 59/63 is 94%.		
R81	IP	20% of 63 patients is impossible.	Table 1	13/63(20%) of radiotherapy patients had pleural or pericardial effusions. 12/63 is 19%. 13/63 is 21%.		
R81	IP	4% of 63 patients is impossible.	Table 1	3/63(4%) of radiotherapy patients had performance status 0. 2/63 is 3%. 3/63 is 5%.		
R81	IP	61% of 63 patients is impossible.	Table 1	39/63(61%) of radiotherapy patients have performance status 1. 38/63 is 60%. 39/63 is 62%.		
R81	IP	55% of 61 patients is impossible.	Table 1	23/61 (55%) of non-radiotherapy patients were male. 33/61 is 54%. 34/61 is 56%.		
R81	IP	14% of 61 patients is impossible.	Table 1	9/61 (14%) of no radiotherapy patients had low international project index. 8/61 is 13%. 9/61 is 15%.		
R81	IP	96% of 61 patients is impossible.	Table 1	59/61(96%) of no radiotherapy patients had bulky disease. 58/61 is 95%. 59/61 is 97%.		
R81	IP	63% of 61 patients is impossible.	Table 1	40/61(63%) of no radiotherapy patients is not possible. 38/61 is 62%. 39/61 is 64%.		
R81	PP	124/158 patients is not 98%	Results	Complete response was achieved in 124 cases (98%). 124/158 is 78%. 98% is possible but arises from 155/158.	Results	From June 2002 to June 2004, 158 patients were enrolled in the study. In this intent-to-treat population, all cases were available for efficacy and toxicity tests.
R81	PP	45/(158*6) is not 6%.	Results page 1229	Granulocytopenia observed in 45 cycles (6%). 45/(158*6) is 5%. 6% is possible but arises from between 53 and 61/(158*6).	Results	158 patients were enrolled. All patients received the planned six cycles of treatment.
R81	PP	11/(158*6) is not 1.6%.	Results page 1229	Infection-related granulocytopenia in 11 cycles (1.6%). 11/(158*6) is 1.2%. 1.6% is possible but arises from 15/(158*6).	Results	158 patients were enrolled. All patients received the planned six cycles of treatment.
R81	PP	35/63 patients is not 37%.	Table 1	35/63(37%) of radiotherapy group female. 35/63 is 56%. 37% is possible but arises from 23/63.		
R81	PP	38/61 patients is not 61%.	Table 1	38/61(61%) of no radiotherapy group were female. 38/61 is 62%. 60% is possible but arises from 37/61.		
R81	PP	48/61 patients is not 77%.	Table 1	48/61(77%) of no radiotherapy had Stage II disease. 48/61 is 79%. 77% is possible but arises from 47/61.		
R81	PP	7/61 is not 13%.	Table 1	7/61(13%) of no radiotherapy group had pleural or pericardial effusions. 7/61 is (11%). 13% is possible but arises from 8/61.		
R82	FD	Contradiction between KM diagram and number of exacerbations.	Fig 3A	13/18 placebo had an asthma exacerbation.	Fig 3B	11 step downs on diagram (10 single and one double)
R82	FD	Total asthma symptom score. There is no consistent definition of "baseline" measurement value, i.e. which time points to average, that allows both the infliximab and control values to agree between abstract/text and Figure.	Table 1 and Results text, and Figure 2	Infliximab: Mean given as 8.4. Figure 2 shows that the initial value is clearly less than 8.3. The only way of these being reconciled is that it is not the initial value, but some sort of average that is used as the baseline.	Table 1 and Results text, and Figure 2	Placebo: Mean given as 9.6. Figure 2 shows that only the initial value is 9.6. Averaging over a longer time window, which is essential to justify the value for Infliximab, cannot ever produce an average of 9.6 as timepoints after the initial one have lower values.
R82	IP	6.8% of 15 patients is impossible.	Table 1	1(6.8%) Black African in Infliximab PP group. 1/15 is 6.7%. 2/15 is 13.3%.		
R82	IP	6.8% of 15 patients is impossible.	Table 1	1(6.8%) Asian in Infliximab PP group. 1/15 is 6.7%. 2/15 is 13.3%.		
R83	IP	15.3% of 13 patients is impossible.	Results (Page 195)	Two (15.3%) had symptomatic DVT. 1/13 is 7.7%. 2/13 is 15.4%.		
R83	IP	74% of 60 patients is impossible.	Results (Page 195)	The overall response rate was 74%. 44/60 patients is 73%. 45/60 patients is 75%.		
R83	IP	24% of 60 patients is impossible.	Results (Page 195)	24% had a complete remission. 14/60 patients is 23%. 15/60 patients is 25%.		
R83	IP	16% of 60 patients is impossible.	Results (Page 195)	16% failed to respond. 9/60 is 15%. 10/60 is 17%.		
R83	IP	96% of 60 patients is impossible.	Results (Page 195)	96% proceeded to PBSC mobilization. 57/60 is 95%. 58/60 is 97%.		
R83	ISS	Impossible subgroups (for age) medians for PLTC>50,000/ul (days)	Table 4	Median for >=50 years is 19. Median for <50 years is 18.	Table 3	Median for all ages 17 days.

R84	FD	Contradiction over median time to analgesia in Group 1	Results page 397	Median time 160 mins	Figure 1	Median appears to be 120 mins
R84	FD	Contradiction over median time to analgesia in Group 2	Results page 397	Median time 385 mins	Figure 1	Median appears to be 220 mins
R84	FD	Contradiction over median time to analgesia in Group 3	Results page 397	Median time 454 mins	Figure 1	Median appears to be 160 mins
R84	FD	Contradiction over median time to analgesia in Group 4	Results page 397	Median time 974 mins	Figure 1	Median appears to be 230 mins
R85	IP	3.9% of 292 patients is impossible.	Table 2	11/292(3.9%) in home users had incomplete abortion. 11/292 is 3.8%. 12/292 is 4.1%.		
R85	IP	0.6% of 292 patients is impossible.	Table 2	2/292(0.6%) in home users had medically indicated surgical abortion. 1/292 is 0.3%. 2/292 is 0.7%.		
R85	IP	8.3% of 323 patients is impossible.	Table 2	27/323(8.3%) in home users had no evidence either way. 26/323 is 8.0%. 27/323 is 8.4%.		
R85	IP	2.4% of 367 patients is impossible.	Table 2	9/367(2.4%) in all users had ongoing pregnancy. 8/367 is 2.2%. 9/367 is 2.5%.		
R86	AE	2 patients >65 are not accounted for in treatment or control arm.	Table 1	94 over 65s in treatment arm + 91 over 65s in control arm is 185. 187 >65s in total in the trial.		
R86	AE	10 patients with abdominal disease are not accounted for in treatment of control arm.	Table 2	37 abdomen in treatment arm + 29 abdomen in control arm is 66 not 76 in total.		
R86	PP	2/1467 patients is 0% or 0.1%, not 1%	Results Safety Pulmonary Fibrosis page 1299	Pulmonary fibrosis occurred in 1% (2 of 1,467). 2/1467 is 0.136% which rounds to 0% or 0.1%. 1% still possible but arises from between 8 and 22/1,467.		
R86	PP	94/231 patients is not 42%.	Table 1	94/231(42%) of panitumumab plus BMC group were >=65. 94/231 is 41%. 42% still possible but arises from between 96 and 98/231.		
R86	PP	29/232 patients is not 17%.	Table 2	29/232(17%) of BSC alone group had abdominal disease. 29/232 is 13%. 17% is possible but arises from 39 or 40/232.		
R86	PP	71/232 patients is not 30%.	Table 2	71/232 (30%) of BSC alone group had >35% cells staining EGFR positive. 71/232 is 31%. 30% is still possible but arises from 69 or 70/232.		
R87	FD	Relative risk value shown contradicts directly calculated value	Statement in Figure 2	Relative risk for TAC versus FAC given as 0.57	Information contained in Figure 2.	Actual relative risk = risk in TAC / risk in FAC = (2/34)/(3/27) = 0.53
R87	FD	Contradiction over number of % in group	Table 2	n=34 in TAC group	Table 3	TAC described as 34%
R87	FD	Contradiction over number or % in group	Table 2	n=27 in FAC group	Table 3	FAC described as 27%
R87	FD	Contradiction over % of FAC patients with (specifically) febrile neutropenia	Table 3	Proportion given as 26%	Results (Page 168)	Proportion given as 76%
R87	FD	Contradiction over where DFS compared	Text and Figure 1 legend	33 months	Figure 1	36 months
R87	FD	Contradiction over where OS compared	Text and Figure 2 legend	33 months	Figure 2	36 months
R87	IP	45% of 27 patients is impossible.	Table 2	45% of FAC were ER+ and/or PR+. 12/27 is 44%. 13/27 is 48%.		
R88		none				
R89		none				
R90	PP	8/30 patients is not 23%.	Table 2	8/30(23%) in placebo group vomited. 8/30 is 27%. 23% is possible but arises from 7/30.		
R90	PP	7/30 patients is not 20%.	Table 2	7/30(20%) in propofol 0.25mg/kg group vomited. 7/30 is 23%. 20% is possible can arises from 6/30.		
R90	PP	3/30 patients is not 7%.	Table 2	3/30(7%) in propofol 0.5mg/kg group vomited. 3/30 is 10%. 7% is possible but arises from 2/30.		
R91	FD	Contradiction over % of vomiting that was severe at Hospital B	Results	32% at Hospital B	Table 2	31% at Hospital B
R91	FD	Contradiction over number of patients interviewed at Hospital A.	Table 1	4152+1556=5708	Table 2	5749
R91	FD	Contradiction over number of patients interviewed at Hospital C.	Table 1	3456+988=4444	Table 2	4436
R91	FD	Contradiction over number of patients interviewed at Hospital D.	Table 1	1994+1219=3213	Table 2	3212
R91	FD	Contradiction over number of patients interviewed overall.	Table 1	4320+1388+1992+603+3486+958+2954+259=15960	Abstract	16000 interviews
R91	PP	999/3212 is not 31.3%.	Table 1	Hospital D: chart review no, interview yes. 999/3212 is not 31.3%		
R91	PP	220/3212 is not 6.9%.	Table 1	Hospital D: chart review yes, interview yes. 220/3212 is not 6.9%		
R91	PP	588/959 is not 62%.	Table 2	Hospital A: vomiting that was severe. 588/959 is 61% not 62%.		
R91	PP	918/4436 is not 22%.	Table 2	Hospital C: proportion with nausea. 918/4436 is 21% not 22%.		
R91	PP	1182/3212 is not 38%.	Table 2	Hospital D: proportion of patients with nausea. 1182/3212 is 37% not 38%		

R92	PP	Incorrect calculation of required sample size	Methods - Statistical Analysis (Page 1273)	For 90% power 0.05 alpha, mean difference 2.9, SD 1.0 and four groups, the authors report calculating the need for 16 patients per group.		When calculated by standard methods the number needed is only 2 per group, i.e. 8 total. This is because the planned effect size (mean difference divided by SD) is so large, at 2.9.
R92	PP	% of Pregabalin group with light-headedness or dizziness calculated incorrectly	Table 4	4/20 is 20% not 25%. 25% is possible but arises from 5/20.		
R93	AE	Smoking Status of PCI arm do not add up to 100%	Table 1	72% (ex-smoker) + 12%(never-smoker) +13%(current smoker) = 97%		
R93	AE	Smoking Status of PC arm do not add up to 100%	Table 1	66% (ex-smoker) + 12% (never-smoker) + 20%(current smoker) = 98%		
R93	FD	Contradiction over number of patients in PCI20 arm	Abstract	50 patients received PCI20	Figure 1	53 patients received PCI20
R93	FD	Contradiction over number of smokers in study.	Table 1	20% in the PC group (=10.6) and 13% in the PCI group (=12.74) together are at most 24 patients (based on11+13)	Results page 2520	n=25 current smokers.
R93	FD	Contradiction over numbers of never-smokers together in both groups	Table 1	12 % in the PC group (=6.36 patients) and 12% in the PCI group (=11.76 patients) together are more than 17 patients	Results page 2520	n=17 never smokers
R93	IP	52% of 53 patients is impossible.	Results (Treatment)	52% of PCI20 cohort were adenocarcinoma. 27/53 is 51%. 28/53 is 53%		
R93	IP	18% of 53 patients is impossible.	Results (Treatment)	18% of PCI20 cohort were squamous cell. 9/53 is 17%. 10/53 is 19%.		
R93	IP	20% of 53 patients is impossible.	Table 1	20% of 53 in PC arm are smokers. 10/53 is 19%. 11/53 is 21%.		
R93	IP	80% of 53 patients is impossible.	Table 1	80% in PC arm had Stage IV disease. 42/53 is 79%. 43/53 is 81%.		
R93	IP	25% of 98 patients is impossible.	Table 1	25% in PC arm had histological subtype NOS. 24/98 is 24%. 25/98 is 26%.		
R93	IP	12% of 53 patients is impossible.	Table 1	12% in PC arm were never-smokers. 6/53 is 11%. 7/53 is 13%.		
R93	IP	7% of 53 patients is impossible.	Table 4	7% of PC arm had Grade IV neutropenia. 3/53 is 6%. 4/53 is 8%.		
R93	IP	1% of 53 patients is impossible.	Table 4	1% of PC arm had Grade IV thrombocytopenia. 0/53 is 0%. 1/53 is 2%.		
R93	IP	1% of 53 patients is impossible.	Table 4	1% of PC arm had Grade IV infection. 0/53 is 0%. 1/53 is 2%.		
R94	AE	The mutually-exclusive antibiotic strategies in study group add up to more than patient numbers	Table III	n=39 in study group	Table III	Different antibiotic strategies were Keflin (24h) in 21, Keflin + Gentamicin in 10 and Keflin (48h) in 9. 21+10+9=40
R94	FD	Contradiction over size of study group	Table I	n=39 in Study Group	Table II	n=30 in Study Group
R94	IP	48.71% of 39 patients is impossible.	Table III	19/39 (48.71%) had hospital stay less than 4 days in study group. 18/39 is 46.2%. 19/39 is 48.7%.		
R94	IP	41.02% of 39 patients is impossible.	Table III	16/39 (41.02%) had hospital stay 4-6 days in study group. 15/39 is 38.5%. 16/39 is 41.0%.		
R94	IP	10.25% of 39 patients is impossible.	Table III	4/39 (10.25%) had hospital stay more than 6 days in study group. 3/39 is 7.69%. 4/39 is 10.26%.		
R94	IP	22.3% of 27 patients is impossible.	Table III	6/27 (22.3%) had 48h Keflin in control group. 6/27 is 22.22%. 7/27 is 25.93%.		
R94	IP	44.24% of 27 patients is impossible.	Table III	12/27 (44.24%) had hospital stay less than 4 days in control group. 11/27 is 40.74%. 12/27 is 44.44%.		
R94	PP	13/39 is not 30.8%.	Table I	13/39 (30.8%) of study group had hypertension. 13/39 is 33.3%. 30.8% is possible but arises from 12/39.		
R94	PP	3/39 is not 10.3%.	Table I	3/39 (10.3%) had "Others" in study group. 3/39 is 7.7%. 10.3% is possible but arises from 4/39.		
R94	PP	9/39 is not 20.5%.	Table III	9/39(20.5%) had Keflin(48h) in study group. 9/39 is 23.1%. 20.5% is possible but arises from 8/39.		
R95		none				
R96	FD	Contradiction over participants rating ALC scheme milder	Results	0.8	Figure 2	17/20 rated ALC milder (85%)
R96	FD	Contradiction over proportion assigned to each group	Results, Methods	"Fifty subjects entered the study; half each in the ALC and CHG groups." "25 subjects were recruited into each treatment group"	Table 1	Characteristics indicate 26 in ALC group and 24 in CHG group
R96	IP	62.2% of 69 observations is impossible.	Results page 948 observations in medical ICU	42/69 is 60.9%. 43/69 is 62.3%. 69 observations were undertaken in total (36 in medical ICU, 33 in surgical ICU).		
R96	ISS	Contradiction over mean handwashes per shift	Results	11.7	Table 2	26*6.1 and 24*16.7 is 11.2 (doesn't work even with rounding)
R96	ISS	Contradiction over average number of hours in gloves	Results	2.6	Table 1	26*2.15 and 24*2.95 is 2.53 (so doesn't work even with rounding)
R97		none				
R98	FD	Contradiction over mean base excess at baseline in the balanced group	Abstract Text	BE is 1.04	text page 465	BE is 1.16
R98	ISS	Median ICU length of stay outside range of length of stay for unbalanced group	Table 1	Median LOS 13 days	Table 1	LOS Range (14-444 days)
R98	ISS	Range of cross-clamp time is impossibly narrow for that standard deviation.	Table 1	55±14 (45–70). Even if all data is extremes (e.g. 12 at 44.5 and 13 at 70.5) the standard deviation is 13.26 which cannot be rounded to 14.		
R99	AE	% of Methylated Patients adds up to 122%. Legend confirms the numbers in the table are percentages.	Table 1	57% were younger than 15 years, 65% were over 15 years		

R99	FD	Contradiction over number of male patients	Methods	154 male patients	Table I	60% of 100 patients (nonmethylated) = 60. 61% of 161 patients (methylated) = 98. Total = 158
R99	FD	Contradiction over number of female patients	Methods	107 female patients	Table I	40% of 100 patients (nonmethylated) = 40. 39% of 161 patients (methylated) = 63. Total = 103
R99	FD	Contradiction over number of relapses	Methods	Relapses occurred in 106 patients.	Table I	29% of 100 patients (nonmethylated) = 29. 59% of 161 patients (methylated) had relapse = 95. Total = 124.
R99	FD	Discrepancy in percentage of patients being either younger or older than 15 years exceeding 100% in the methylated group	Table I	57% plus 65% exceeds 100%. Note footnote of the Table ("Data are expressed as percentages of patient population, except where otherwise noted.")		
R99	ISS	Stated median age contradicts the descriptions of the ranges in the two subgroups: the larger subgroup must contain the median.	Methods page 3463	Describing age at diagnosis: "Of these patients, 129 were children (median age, 5 years; range, 0.5-14 years) and 132 presented adult ALL (median age, 29 years; range, 15-82 years)", which shows that the majority were aged 15 or above. Therefore the median age MUST be at least 15.	Methods page 3463	"The median age at diagnosis in the study population as a whole was 14 years (range, 0.5-82 years)."
R100	FD	Discrepancy in the number of patients in the IVA group who received piritramide postoperatively	Table IIB	Number of patients stated to be 24+29+26=79	Table IIC	Number of patients stated to be 78
R100	FD	Contradiction over number of patients in Isoflurane group with PONV (24h)	Table IIC	59 (first row)	Table IIC	16 (fourth row)
R100	FD	Contradiction over number of patients in Desflurane group with PONV (24h)	Table IIC	47 (first row)	Table IIC	20 (fourth row)
R100	FD	Contradiction over number of patients in IVA group with PONV (24h)	Table IIC	36 (first row)	Table IIC	15 (fourth row)
R100	IP	1.5% of 129 patients is impossible.	Results (page 1025).	Two patients retched (1.5%) (from group of 129). 1/129 is 0.8%. 2/129 is 1.6%.		
R100	IP	5.5% of 129 patients is impossible.	Table IIA	Number of patients with PONV (4-24 hr) 3rd row Group D. 7/129 is 5.4%. 8/129 is 6.2%.		
R100	IP	11.5% of 129 patients is impossible	Table IIC	Number of patients with PONV (0-24 hr). IVA 4th row. 14/129 is 10.9%. 15/129 is11.6%.		
R100	PP	40/129 is not 41%.	Table IIC	Number of patients who received piritramide is 40/129(41%).		
