# Supplementary material for: Frequency of discrepancies in retracted clinical trial reports versus unretracted reports: blinded case-control study
Source: BMJ. 2015 Sep 20;351:h4708. doi: 10.1136/bmj.h4708 (PMC4575810; doi:10.1136/bmj.h4708)

Web appendix 4

Online Figure 1 - For the subgroup of 68 trial reports meeting the criteria of being a trial of an intervention.

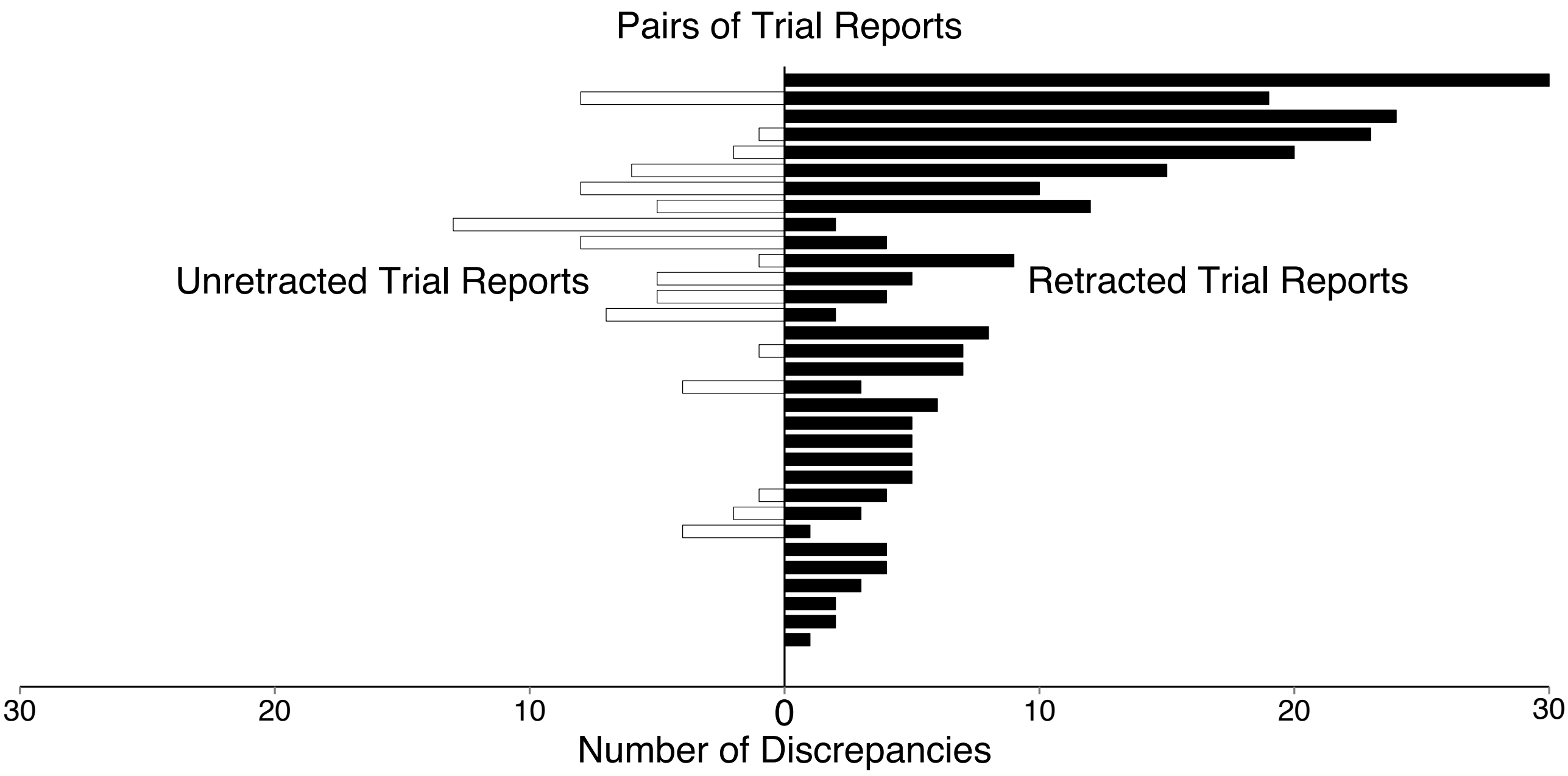

Online Figure 2 - For the subgroup of 68 trial reports meeting the criteria of being a trial of an intervention.

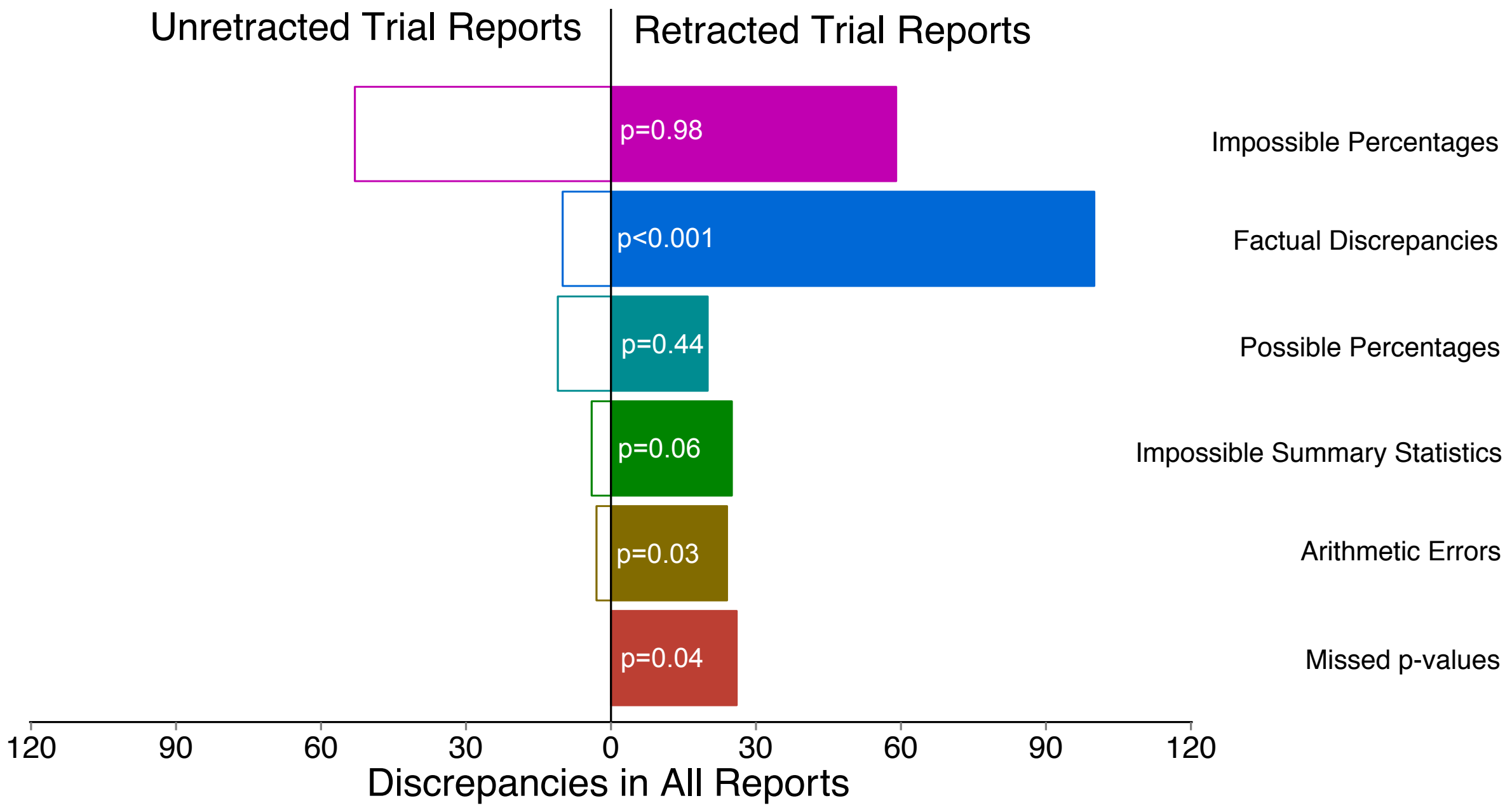

Online Figure 3 - For the subgroup of 68 trial reports meeting the criteria of being a trial of an intervention.

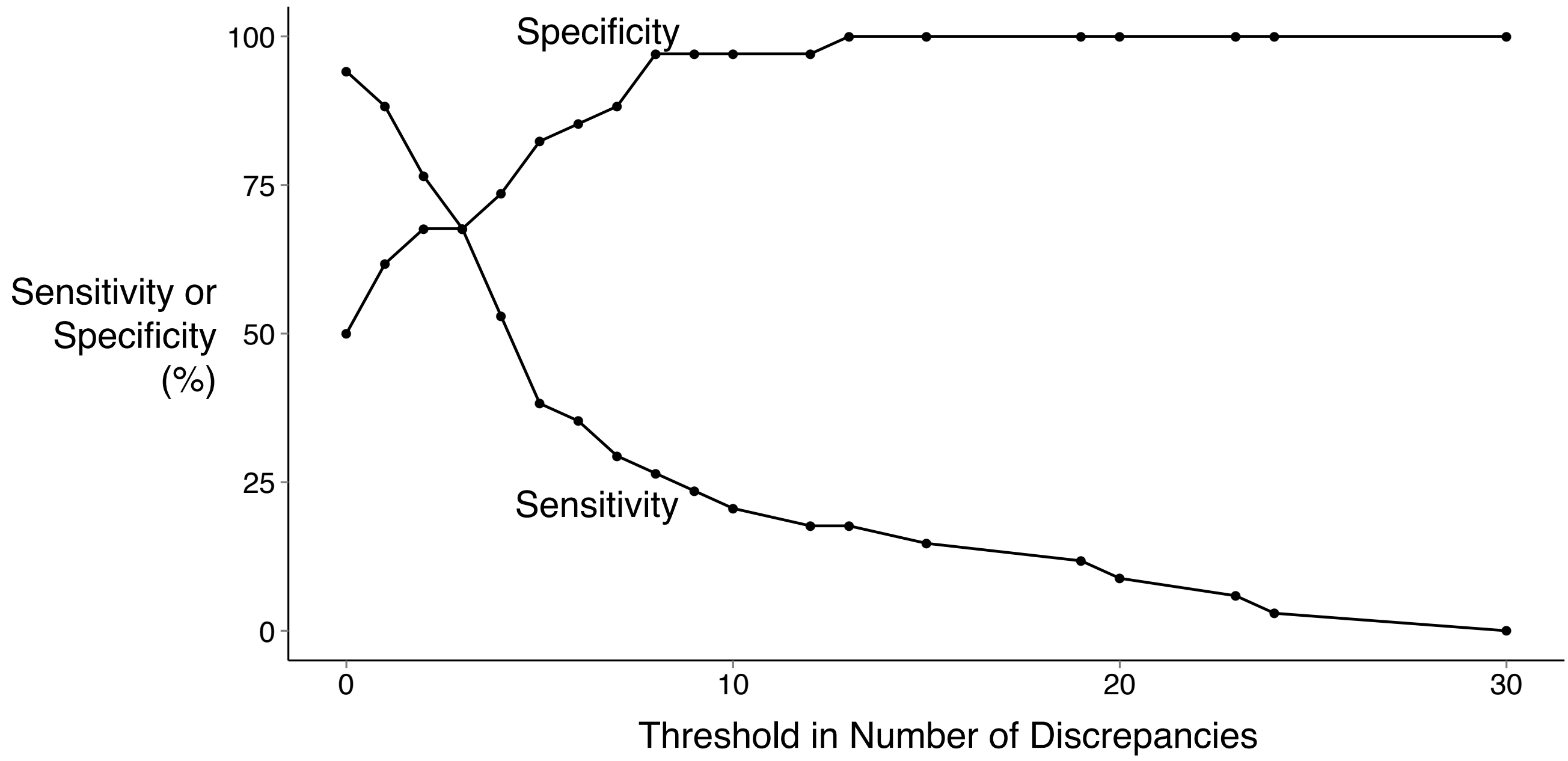

Supplement: Supplementary file 4 [file colg020413.ww4_default.pdf]
