# Supplementary material for: Frequency of discrepancies in retracted clinical trial reports versus unretracted reports: blinded case-control study
Source: BMJ. 2015 Sep 20;351:h4708. doi: 10.1136/bmj.h4708 (PMC4575810; doi:10.1136/bmj.h4708)
Supplement: Supplementary file 5 [file colg020413.ww5_default.pdf]

Web appendix 5

For the subgroup of 68 trial reports meeting the criteria of being a trial of an intervention.

Univariable Analysis

**Binomial Component**	**Incidence Rate Ratio**	**95% Confidence Interval**	**p-value**
Intercept	4.07	(2.51 to 6.59)	<0.001
Retraction	1.84	(1.04 to 3.26)	0.04

**Excess Zero Component**	**OR**	**95% Confidence Interval**	**p-value**
Intercept	0.71	(0.30 to 1.69)	0.43
Retraction	0.000	(0.000 to inf)	1.00

Multivariable Analysis

**Binomial Component**	**Incidence Rate Ratio**	**95% Confidence Interval**	**p-value**
Intercept	1.48	(0.49 to 4.40)	0.49
Retraction	2.00	(1.15 to 3.46)	0.01
Year	1.06	(1.00 to 1.13)	0.04

**Excess Zero Component**	**OR**	**95% Confidence Interval**	**p-value**
Intercept	71.6	(1.42 to 3612)	0.03
Retraction	0.007	(0.000 to 13.45)	0.20
Year	0.72	(0.54 to 0.97)	0.03
