# Supplementary material for: Frequency of discrepancies in retracted clinical trial reports versus unretracted reports: blinded case-control study
Source: BMJ. 2015 Sep 20;351:h4708. doi: 10.1136/bmj.h4708 (PMC4575810; doi:10.1136/bmj.h4708)
Supplement: Supplementary file 6 [file colg020413.ww6_default.pdf]

Web appendix 6

pair	R-code	discrep	retraction	year	if_current	if_then	citations	actually_trial	op1_mins	op2_mins	op3_mins	total_mins	factual_discrepancies	missed_p_values	impossible_summary_statistics	impossible_percentages	possible_percentages	arithmetic_errors
1	44	15	0	2007	2.809	2.859	41	0	50	43		93	11	0	1	3	0	0
2	45	0	0	2011	4.176	4.524	7	1	41	43		84	0	0	0	0	0	0
3	80	8	0	2010	3.011	1.679	17	1	27	22	12	61	0	0	0	8	0	0
4	89	0	0	2000	4.227	2.913	66	1	51	40	20	111	0	0	0	0	0	0
5	12	1	0	2012	2.818	1.432	13	1	13	35	9	57	0	0	0	1	0	0
6	57	2	0	2009	4.295	3.958	5	1	28	45		73	0	0	0	1	0	1
7	86	6	0	2008	8.193	6.488	37	1	36	27		63	0	0	0	0	4	2
8	15	8	0	2001	1.537	2.871	14	1	12	123	5	140	1	0	2	3	2	0
9	3	5	0	2004	3.422	2.18	7	1	31	26	14	71	0	0	0	2	3	0
10	47	13	0	2010	3.422	3.274	5	1	37	16		53	1	0	0	12	0	0
11	72	0	0	2009	17.96	17.793	69	0	48	32		80	0	0	0	0	0	0
12	52	0	0	2010	3.422	3.274	15	0	34	71	3	108	0	0	0	0	0	0
13	60	8	0	2010	1.2	1.116	3	1	35	38	20	93	0	0	0	8	0	0
14	67	1	0	2008	3.422	2.59	22	1	32	18	8	58	0	0	0	0	1	0
15	35	3	0	2005	13.246	8.016	40	1	23	11	46	80	1	0	1	0	0	1
16	96	5	0	2001	6.147	3.468	84	1	19	13	10	42	2	0	2	1	0	0
17	8	10	0	1994	3.422	2.698	19	0	26	479	30	535	0	0	10	0	0	0
18	91	10	0	1999	4.354	2.387	211	1	35	11	35	81	5	0	0	0	5	0
19	97	0	0	2010	1.21	1.279	2	0	23	61		84	0	0	0	0	0	0
20	9	5	0	2006	11.968	9.091	236	1	31	38		69	1	0	0	3	1	0
21	28	7	0	2006	4.585	3.769	19	1	42	94	14	150	0	0	0	7	0	0
22	13	0	0	2002	2.495	3.24	4	1	26	14	4	44	0	0	0	0	0	0
23	5	1	0	2003	1.806	1.544	7	1	11	120	10	141	0	0	0	1	0	0
24	23	0	0	2008	9.775	10.432	190	1		8		8	0	0	0	0	0	0
25	85	4	0	2009	1.563	1.41	12	1	30			30	0	0	0	4	0	0
26	49	0	0	2007	9.775	10.896	40	0	35	6	3	44	0	0	0	0	0	0
27	10	0	0	2005	3.466	2.643	40	1	42	9	3	54	0	0	0	0	0	0
28	24	6	0	2008	2.551	2.342	7	1	21	12	13	46	6	0	0	0	0	0
29	34	0	0	2000	3.422	2.321	208	1	49	114	21	184	0	0	0	0	0	0
30	88	0	0	2008	30.387	31.718	9	1	30	64	4	98	0	0	0	0	0	0
31	19	0	0	1995	54.42	22.412	21	1	21	10	8	39	0	0	0	0	0	0
32	2	0	0	2003	3.143	1.714	260	1	40	27	10	77	0	0	0	0	0	0
33	54	1	0	2008	9.213	6.444	11	1	62	41	15	118	0	0	0	1	0	0
34	26	2	0	2007	3.1	2.795	33	1	41	55	18	114	1	0	0	1	0	0
35	84	4	0	2006	3.422	2.131	4	1	21	25		46	4	0	0	0	0	0
36	76	5	0	2008	1.563	3.566	10	0	22	97	18	137	0	0	0	3	2	0
37	6	0	0	2000	3.846	2.027	22	1	40	18	12	70	0	0	0	0	0	0
38	22	0	0	1999	3.846	1.879	3	1	38	13	5	56	0	0	0	0	0	0
39	14	1	0	2007	39.207	28.638	113	0	26	105	19	150	0	0	0	0	1	0
40	95	0	0	2009	5.544	5.168	28	1	25	31	4	60	0	0	0	0	0	0
41	18	0	0	2009	3.739	2.934	6	0	40	25	2	67	0	0	0	0	0	0
42	78	0	0	2004	2.737	2.187	45	0	22	14	17	53	0	0	0	0	0	0
43	68	0	0	1994	3.736	2.609	4	1	37	9	6	52	0	0	0	0	0	0
44	30	0	0	2006	3.422	2.131	214	1	41	12	3	56	0	0	0	0	0	0
45	40	0	0	2008	1.008	0.94	12	0		28	14	42	0	0	0	0	0	0
46	41	0	0	1998	4.354	2.34	20	1		20	4	24	0	0	0	0	0	0
47	51	0	0	2000	28.054	27.905	22	0	25	40	5	70	0	0	0	0	0	0
48	62	0	0	1999	2.932	1.916	281	1	50	44		94	0	0	0	0	0	0
49	70	0	0	1998	4.354	2.34	16	1	41	10	4	55	0	0	0	0	0	0
50	59	0	0	2005	2.871	2.054	22	0	48	13	3	64	0	0	0	0	0	0
1	27	35	1	2007	2.809	2.859	29	0	43	62		105	1	0	0	32	2	0
2	81	30	1	2011	4.176	4.524	5	1	45	107		152	7	0	0	11	7	5
3	79	19	1	2010	3.011	1.679	6	1	32	144	7	183	15	0	0	1	0	3
4	21	24	1	2000	4.227	2.913	26	1	31	106	30	167	18	0	0	0	0	6
5	7	23	1	2012	2.818	1.432	5	1	23	12	5	40	13	0	10	0	0	0
6	46	20	1	2009	4.295	3.958	25	1	41			41	2	0	0	13	0	5
7	32	15	1	2008	8.193	6.488	23	1	48	43		91	0	1	0	14	0	0
8	94	10	1	2001	1.537	2.871	1	1	41	44	25	110	1	0	0	5	3	1
9	31	12	1	2004	3.422	2.18	39	1	36	79	4	119	0	12	0	0	0	0
10	66	2	1	2010	3.422	3.274	1	1	24	16		40	0	0	2	0	0	0
11	93	14	1	2009	17.96	17.793	171	1	27	65		92	3	0	0	9	0	2
12	55	13	1	2010	3.422	3.274	10	1	40	7	5	52	10	0	0	0	0	3
13	71	4	1	2010	1.2	1.116	13	1	34	11	11	56	0	3	0	0	0	1
14	17	9	1	2008	3.422	2.59	40	1	39	37	3	79	9	0	0	0	0	0
15	39	7	1	2005	13.246	8.016	31	0	26	80	16	122	0	7	0	0	0	0
16	64	5	1	2001	6.147	3.468	29	1	22	13	5	40	4	0	1	0	0	0
17	69	0	1	1994	3.422	2.698	33	0	32	20	2	54	0	0	0	0	0	0
18	11	0	1	1999	4.354	2.387	5	0	24	10	3	37	0	0	0	0	0	0
19	16	9	1	2010	1.21	1.279	2	1	29	46		75	1	0	0	7	1	0
20	82	4	1	2006	11.968	9.091	153	1	17	28		45	2	0	0	2	0	0
21	37	2	1	2006	4.585	3.769	4	1	21	19	4	44	0	2	0	0	0	0
22	100	8	1	2002	2.495	3.24	18	1	26	75	15	116	4	0	0	3	1	0
23	87	7	1	2003	1.806	1.544	7	1	28	16	9	53	6	0	0	1	0	0
24	58	7	1	2008	9.775	10.432	77	1	22	90		112	2	0	0	2	3	0
25	90	3	1	2009	1.563	1.41	7	1	18	18		36	0	0	0	0	3	0
26	99	6	1	2007	9.775	10.896	113	0	21	37	21	79	4	0	1	0	0	1

27	83	6	1	2005	3.466	2.643	27	1	24	77	9	110	0	0	1	5	0	0
28	61	0	1	2008	2.551	2.342	133	0	40	21	4	65	0	0	0	0	0	0
29	74	5	1	2000	3.422	2.321	15	1	37	283	17	337	4	0	0	0	0	1
30	25	5	1	2008	30.387	31.718	104	1	26	7	4	37	0	5	0	0	0	0
31	36	5	1	1995	54.42	22.412	340	1	32	47	8	87	0	0	5	0	0	0
32	73	5	1	2003	3.143	1.714	6	1	28	31	2	61	3	0	2	0	0	0
33	50	4	1	2008	9.213	6.444	24	1	24	13	3	40	2	0	0	2	0	0
34	53	3	1	2007	3.1	2.795	2	1	34	25	5	64	3	0	0	0	0	0
35	38	1	1	2006	3.422	2.131	29	1	21			21	0	0	0	0	0	1
36	33	0	1	2008	1.563	3.566	10	1	44	11	5	60	0	0	0	0	0	0
37	29	4	1	2000	3.846	2.027	8	1	13	60	8	81	2	0	2	0	0	0
38	75	4	1	1999	3.846	1.879	33	1	28	26	6	60	1	3	0	0	0	0
39	4	3	1	2007	39.207	28.638	86	1	22	65	5	92	3	0	0	0	0	0
40	98	3	1	2009	5.544	5.168	31	1	15	92	7	114	1	0	2	0	0	0
41	56	3	1	2009	3.739	2.934	7	1	22	34	3	59	3	0	0	0	0	0
42	77	3	1	2004	2.737	2.187	51	1	20	26	11	57	1	0	0	1	1	0
43	92	2	1	1994	3.736	2.609	18	1	13	46	8	67	0	0	0	0	2	0
44	42	2	1	2006	3.422	2.131	57	1	41	11	9	61	1	0	0	0	1	0
45	43	1	1	2008	1.008	0.94	7	1	38	14	5	57	1	0	0	0	0	0
46	63	1	1	1998	4.354	2.34	13	1		27	4	31	0	0	0	0	0	1
47	20	0	1	2000	28.054	27.905	542	1	35	15	4	54	0	0	0	0	0	0
48	48	0	1	1999	2.932	1.916	16	1	44	65		109	0	0	0	0	0	0
49	1	0	1	1998	4.354	2.34	35	1	17	22	6	45	0	0	0	0	0	0
50	65	0	1	2005	2.871	2.054	25	0	28	8	3	39	0	0	0	0	0	0
